# Supplementary material for: Cord Blood Metabolite Profiles and Their Association with Autistic Traits in Childhood
Source: Metabolites. 2023 Nov 9;13(11):1140. doi: 10.3390/metabo13111140 (PMC10672851; doi:10.3390/metabo13111140)
Supplement: Supplementary file 1 [file metabolites-13-01140-s001.zip › metabolites-2663432-supplementary.pdf]

Supplementary Material

Supplementary Online Content

Supplemental Table S1. Non-response analysis in singleton live births with and without outcome measurements.

Supplemental Table S2. Cord blood metabolite concentrations (N = 783).

Supplemental Table S3. Associations of cord blood individual metabolites and metabolite groups with SRS scores at age 6 and 13. Basic model.

Supplemental Table S4. Associations of cord blood individual metabolites and metabolite groups with SRS scores at ages 6 and 13. Main model.

Supplemental Table S5. Associations of cord blood individual metabolites and metabolite groups with SRS scores at ages 6 and 13. Adjusted model.

Supplemental Table S6. Associations of cord blood metabolite ratios with SRS scores at ages 6 and 13. Basic model.

Supplemental Table S7. Associations of cord blood metabolite ratios with SRS scores at ages 6 and 13. Main model.

Supplemental Table S8. Associations of cord blood metabolite ratios with SRS scores at ages 6 and 13. Adjusted model.

Supplemental Table S9. P-values of interaction effects of linear mixed-effect models.

Supplemental Table S10. Parameters for mass-spectrometry detection and identifications

Supplemental Figure S1. Difference in SRS score at age 13 per SDS increase in cord-blood metabolite concentration

Supplemental Text S1. Metabolite measurements.

This supplementary material has been provided by the authors to give readers additional information about their work

Supplemental Table S1. Non-response analysis in singleton live births with and without outcome measurements.

	Children included in the analysis (n= 783)	Children not included in the analysis (n= 138)	p-value
**Maternal characteristics**			
Age at enrollment years, mean (±SD)	31.83 (3.88)	29.78 (4.98)	<0.01
Missing (%)	0		
Education level, high, n (%)	510 (66%)	60 (43%)	<0.01
Missing	7 (1%)	0 (0%)	
Parity, n (%)			
Nullipara	490 (62%)	67 (49%)	<0.01
Multipara	293 (38%)	71 (51%)	
Pre-pregnancy body mass index in kg/m^2, median (95% range)	22.38 (18.54, 34.02)	22.68 (18.27, 32.97)	0.64
Smoking, n (%)			
Never smoked during pregnancy	555 (79%)	81 (60%)	<0.01
Smoked until pregnancy was recognized	63 (9%)	16 (12%)	
Continued smoking during pregnancy	84 (12%)	38 (28%)	
Missing	81 (10%)	3 (2%)	
Alcohol use, n (%)			
Never alcohol in pregnancy	207 (30%)	58 (43%)	<0.01
Alcohol until pregnancy was known	103 (15%)	31 (23%)	
Alcohol continued in pregnancy	389 (56%)	46 (34%)	
Missing	85 (11%)	3 (2%)	
Psychopathologies, median, (95% range)	0.12 (0.00, 1.00)	0.13 (0.00, 1.00)	0.07
Folic acid supplement, yes, n (%)	600 (93%)	95 (81%)	<0.01
Missing	137 (17%)	20 (15%)	

Vitamin D deficiency, yes, n (%)	221 (30%)	43 (34%)	0.40
Missing	50 (6%)	13 (10%)	
**Fetal characteristics**			
Fetal sex, female, n (%)	372 (48%)	52 (38%)	0.04
Gestational age at birth in weeks, median (95% range)	40.29 (36.94, 42.43)	40.14 (35.75, 42.22)	0.39
Birthweight in grams, mean (±SD)	3550 (496.00)	3505 (604.31)	0.51
Birthweight <2500 g, n (%)	16 (2%)	8 (6%)	
Birthweight 2500 to 4500 g, n (%)	744 (95%)	125 (91%)	
Birthweight >4500 g, n (%)	23 (3%)	5 (3%)	

SRS: Social Responsiveness Scale. Values presented as mean (± standard deviation (SD), median (interquartile range (95% range)), or number of participants (valid %). Differences in subject characteristics between the groups were evaluated using Independent Student T-test and Mann-Whitney U for continuous variables and χ^2 tests for categorical variables

Supplemental Table S2. Cord blood metabolite concentrations (N = 783).

Neonatal metabolite profile	µmol/L, median (95%)
**Amino acids (AA)**	4559.70 (3014.71, 6337.24)
BCAA	475.26 (298.9, 695.87)
AAA	254.38 (162.24, 366.92)
EAA	1459.98 (986.42, 1991.98)
NEAA	2371.41 (1525.62, 3373.66)
**Ala**	585.97 (332.78, 991.64)
**Arg**	74.97 (31.63, 130.81)
**Asn**	56.16 (34.04, 93.81)
**Asp**	40.2 (21.34, 78.55)
**Cit**	13.86 (7.85, 23.96)
**Gln**	441.71 (234.59, 821.19)
**Glu**	195.59 (97.62, 403.82)
**Gly**	323.66 (211.94, 475.99)
**His**	133.14 (78.28, 215.38)
**Ile**	73.55 (42.01, 113.11)
**Leu**	142.1 (84.82, 226.71)
**Lys**	335.61 (219.23, 551.42)
**Met**	32.76 (19.98, 52.74)
**Orn**	123.84 (68.92, 205.85)
**Phe**	104.7 (65.43, 156.56)
**Pro**	191.37 (120.32, 334.64)
**Trp**	74.59 (45.16, 122.55)
**Ser**	158.25 (95.61, 298.74)
**Thr**	270.23 (150.48, 445.86)
**Tyr**	74.83 (46.54, 114.44)
**Val**	258.67 (165.83, 375.7)
**Cys**	16.08 (7.04, 40.46)

**Non-esterified fatty acids (NEFA)**	191.32 (80.28, 408.50)
Saturated NEFA	90.71 (36.88, 189.41)
Mono-unsaturated NEFA	56.54 (20.44, 134.89)
Poly-unsaturated NEFA	44.12 (19.45, 93.88)
**NEFA.14:0**	7.22 (2.36, 17.39)
**NEFA.15:0**	1.06 (0.35, 2.56)
**NEFA.16:0**	67.21 (26.41, 141.04)
**NEFA.17:0**	1.1 (0.47, 2.28)
**NEFA.18:0**	13.73 (3.25, 30.25)
**NEFA.24:0**	0.17 (0.06, 0.31)
**NEFA.26:0**	0.14 (0.05, 0.31)
**NEFA.14:1**	1.55 (0.37, 4.46)
**NEFA.16:1**	10.02 (2.96, 28.71)
**NEFA.17:1**	0.53 (0.14, 1.37)
**NEFA.18:1**	42.89 (16.05, 99.56)
**NEFA.19:1**	0.15 (0.06, 0.33)
**NEFA.20:1**	0.41 (0.16, 0.86)
**NEFA.24:1**	0.17 (0.05, 0.32)
**NEFA.26:1**	0.09 (0.05, 0.16)
**NEFA.16:2**	0.47 (0.17, 1.16)
**NEFA.17:2**	0.03 (0, 0.09)
**NEFA.18:2**	24.07 (8.55, 58.41)
**NEFA.18:3**	2.06 (0.44, 6.03)
**NEFA.20:2**	0.55 (0.22, 1.12)
**NEFA.20:3**	1.79 (0.78, 4.03)
**NEFA.20:4**	8.1 (3.65, 15.91)
**NEFA.20:5**	0.28 (0.09, 0.78)
**NEFA.22:3**	0.13 (0.07, 0.25)
**NEFA.22:4**	0.62 (0.33, 1.18)

**NEFA.22:5**	0.74 (0.35, 1.56)
**NEFA.22:6**	4.2 (1.83, 9.4)
**NEFA.24:2**	0.09 (0.05, 0.18)
**NEFA.24:4**	0.12 (0.06, 0.22)
**NEFA.24:5**	0.1 (0.05, 0.2)
**NEFA.26:2**	0.06 (0.03, 0.1)
**Acyl-alkyl-phosphatidylcholines (PC.ae)**	74.01 (47.44, 129.47)
Saturated PC.ae	12.10 (7.22, 21.14)
Mono-unsaturated PC.ae	8.54 (5.13, 15.96)
Poly-unsaturated PC.ae	52.99 (33.05, 93.64)
**PC.ae.C30:0**	0.23 (0.08, 0.47)
**PC.ae.C32:0**	2.37 (1.3, 4.48)
**PC.ae.C34:0**	0.8 (0.4, 1.77)
**PC.ae.C36:0**	0.54 (0.28, 1.07)
**PC.ae.C38:0**	0.95 (0.44, 1.87)
**PC.ae.C40:0**	7.11 (3.69, 12.99)
**PC.ae.C32:1**	2.08 (1.06, 4.22)
**PC.ae.C34:1**	3.72 (2.04, 7.4)
**PC.ae.C36:1**	1.76 (0.96, 3.38)
**PC.ae.C40:1**	0.68 (0.25, 1.39)
**PC.ae.C42:1**	0.27 (0.11, 0.54)
**PC.ae.C32:2**	0.44 (0.19, 0.95)
**PC.ae.C34:2**	2.43 (1.38, 4.82)
**PC.ae.C34:3**	1.1 (0.57, 2.35)
**PC.ae.C36:2**	0.08 (0.02, 0.18)
**PC.ae.C36:3**	2.02 (1.12, 4.02)
**PC.ae.C36:4**	1.91 (1.01, 3.72)
**PC.ae.C36:5**	8.64 (5.07, 15.86)
**PC.ae.C38:2**	6.45 (3.56, 12.69)
**PC.ae.C38:3**	0.5 (0.15, 1.17)
**PC.ae.C38:4**	1.65 (0.83, 3.2)

**PC.ae.C38:5**	7.55 (4.5, 13.67)
**PC.ae.C38:6**	3.31 (1.97, 6.23)
**PC.ae.C40:2**	0.43 (0.06, 1.27)
**PC.ae.C40:3**	0.46 (0.17, 1.06)
**PC.ae.C40:4**	1.8 (0.92, 3.37)
**PC.ae.C40:5**	1.53 (0.85, 2.92)
**PC.ae.C40:6**	2.16 (1.16, 4.04)
**PC.ae.C42:3**	0.28 (0.08, 0.64)
**PC.ae.C42:4**	0.53 (0.2, 1.08)
**PC.ae.C42:5**	1.1 (0.5, 2.32)
**PC.ae.C42:6**	0.9 (0.44, 1.78)
**Diacyl-phosphatidylcholines (PC.aa)**	754.37 (456.57, 1266.98)
Saturated PC.aa	18.03 (10.46, 32.55)
Mono-unsaturated PC.aa	128.03 (75.83, 241.68)
Poly-unsaturated PC.aa	607.24 (375.07, 1022.96)
**PC.aa.C30:0**	2.8 (1.43, 5.48)
**PC.aa.C32:0**	11.53 (6.25, 21.41)
**PC.aa.C36:0**	1.11 (0.43, 2.16)
**PC.aa.C38:0**	1.48 (0.65, 3.03)
**PC.aa.C40:0**	0.51 (0.21, 1.06)
**PC.aa.C42:0**	0.57 (0.27, 1.15)
**PC.aa.C32:1**	9.92 (4.82, 22.65)
**PC.aa.C34:1**	97.7 (57.53, 184.23)
**PC.aa.C36:1**	19.62 (11.2, 35.31)
**PC.aa.C40:1**	0.22 (0.05, 0.48)
**PC.aa.C30:3**	0.13 (0.06, 0.26)
**PC.aa.C32:2**	0.67 (0.06, 1.98)
**PC.aa.C32:3**	0.3 (0.1, 0.61)
**PC.aa.C34:2**	67.54 (37.97, 130.74)
**PC.aa.C34:3**	2.55 (1.24, 5.36)
**PC.aa.C34:4**	0.42 (0.19, 0.84)

**PC.aa.C34:5**	0.05 (0.01, 0.11)
**PC.aa.C36:2**	39.26 (22.45, 70.09)
**PC.aa.C36:3**	66.62 (34.96, 124.29)
**PC.aa.C36:4**	141.64 (76.42, 241.79)
**PC.aa.C36:5**	4.69 (2.31, 10.5)
**PC.aa.C36:6**	0.24 (0.09, 0.51)
**PC.aa.C38:2**	2.85 (1.08, 5.7)
**PC.aa.C38:3**	42.95 (22.16, 77.88)
**PC.aa.C38:4**	100.53 (59.4, 173.86)
**PC.aa.C38:5**	22.07 (12.7, 40.09)
**PC.aa.C38:6**	69.01 (36.57, 132.27)
**PC.aa.C40:2**	0.14 (0.03, 0.36)
**PC.aa.C40:3**	0.41 (0.14, 0.9)
**PC.aa.C40:4**	2.86 (1.52, 5.57)
**PC.aa.C40:5**	6.73 (3.35, 13.8)
**PC.aa.C40:6**	30.29 (14.44, 58.62)
**PC.aa.C42:5**	0.35 (0.15, 0.64)
**PC.aa.C43:6**	1.71 (0.85, 3.37)
**PC.aa.C44:12**	0.29 (0.15, 0.58)
**Acyl-lysophosphatidylcholines (Lyso.PC.a)**	143.63 (82.90, 227.75)
Saturated Lyso.PC.a	91.52 (54.15, 150.48)
Mono-unsaturated Lyso.PC.a	17.74 (9.13, 32.55)
Poly-unsaturated Lyso.PC.a	32.48 (17.21, 57.26)
**Lyso.PC.a.C14:0**	3.25 (1.53, 5.85)
**Lyso.PC.a.C16:0**	72.5 (42.25, 119.57)
**Lyso.PC.a.C18:0**	15.31 (9.41, 25.72)
**Lyso.PC.a.C16:1**	4.73 (2.25, 8.94)
**Lyso.PC.a.C18:1**	13.04 (6.76, 23.37)
**Lyso.PC.a.C18:2**	11.95 (5.79, 22.74)
**Lyso.PC.a.C18:3**	0.29 (0.09, 0.69)
**Lyso.PC.a.C20:3**	3.88 (1.75, 7.38)

**Lyso.PC.a.C20:4**	13.34 (6.57, 24.91)
**Lyso.PC.a.C20:5**	0.28 (0.08, 0.66)
**Lyso.PC.a.C22:6**	2.47 (1.19, 4.81)
**Alkyl-lysophosphatidylcholines (Lyso.PC.e)**	1.66 (0.85, 2.97)
Saturated Lyso.PC.e	1.38 (0.67, 2.56)
Mono-unsaturated Lyso.PC.e	0.28 (0.11, 0.50)
**Lyso.PC.e.C16:0**	0.55 (0.27, 1.05)
**Lyso.PC.e.C18:0**	0.85 (0.32, 1.65)
**Lyso.PC.e.C18:1**	0.28 (0.11, 0.5)
**Sphingomyelines (SM)**	221.43 (132.78, 376.82)
Mono-unsaturated SM	106.85 (66.70, 181.12)
Poly-unsaturated SM	113.43 (64.97, 205.09)
**SM.a.C30:1**	0.14 (0.04, 0.28)
**SM.a.C32:1**	3.03 (1.65, 5.23)
**SM.a.C33:1**	2.2 (1.17, 3.95)
**SM.a.C34:1**	54.84 (34.14, 96.11)
**SM.a.C35:1**	1.66 (0.91, 3.13)
**SM.a.C36:1**	19.45 (11.19, 32.72)
**SM.a.C37:1**	0.96 (0.43, 1.85)
**SM.a.C39:1**	1.35 (0.64, 2.83)
**SM.a.C41:1**	3.97 (2.12, 7.75)
**SM.a.C42:1**	17.97 (10.4, 31.14)
**SM.a.C43:1**	0.95 (0.49, 1.91)
**SM.a.C32:2**	0.45 (0.22, 0.87)
**SM.a.C34:2**	11.21 (6.09, 20.06)
**SM.a.C36:2**	12.55 (6.69, 23.45)
**SM.a.C36:3**	0.35 (0.13, 0.74)
**SM.a.C38:2**	6.09 (2.92, 14.03)
**SM.a.C38:3**	0.2 (0.07, 0.34)
**SM.a.C39:2**	0.46 (0.17, 0.83)
**SM.a.C40:2**	11.81 (5.29, 25.11)
**SM.a.C40:5**	0.27 (0.12, 0.54)

**SM.a.C41:2**	3.42 (1.66, 6.89)
**SM.a.C42:2**	34.97 (19.17, 64.49)
**SM.a.C42:3**	18.22 (8.67, 35.67)
**SM.a.C42:4**	6.78 (3.65, 12.12)
**SM.a.C42:6**	2.8 (1.52, 5.34)
**SM.a.C43:2**	1.27 (0.61, 2.41)
**SM.a.C44:6**	1.4 (0.58, 2.56)
**SM.e.C36:2**	0.24 (0.11, 0.49)
**SM.a.C38:3**	0.06 (0.02, 0.15)
**SM.a.C40:5**	0.22 (0.1, 0.42)
**Free Carn**	16.31 (9.83, 27.66)
**Acyl-carnitine (Carn.a)**	5.82 (3.51, 10.21)
Short-chain Carn.a	4.45 (2.44, 8.53)
Medium-chain Carn.a	0.51 (0.29, 0.87)
Long-chain Carn.a	0.85 (0.49, 1.31)
**Carn.a.C2:0**	3.73 (1.91, 7.44)
**Carn.a.C3:0**	0.3 (0.16, 0.61)
**Carn.a.C3:0.DC**	0.11 (0.04, 0.33)
**Carn.a.C4:0**	0.14 (0.08, 0.26)
**Carn.a.C5:0**	0.14 (0.06, 0.32)
**Carn.a.C6:0**	0.05 (0.02, 0.12)
**Carn.a.C6:0.OH**	0.04 (0.02, 0.09)
**Carn.a.C8.0**	0.06 (0.02, 0.13)
**Carn.a.C8:1**	0.05 (0.02, 0.11)
**Carn.a.C9:0**	0.02 (0.01, 0.04)
**Carn.a.C10:0**	0.09 (0.04, 0.18)
**Carn.a.C10:1**	0.08 (0.04, 0.14)
**Carn.a.C12.0**	0.1 (0.05, 0.18)
**Carn.a.C14:1**	0.05 (0.02, 0.12)
**Carn.a.C14:2**	0.03 (0.01, 0.07)
**Carn.a.C15:0**	0.04 (0.02, 0.07)
**Carn.a.C16:0**	0.16 (0.09, 0.28)

**Carn.a.C16:0.Oxo**	0.02 (0.01, 0.04)
**Carn.a.C16:1**	0.11 (0.05, 0.2)
**Carn.a.C16:2**	0.03 (0.02, 0.06)
**Carn.a.C18:0**	0.09 (0.05, 0.15)
**Carn.a.C18:1**	0.09 (0.05, 0.16)
**Carn.a.C18:2**	0.07 (0.03, 0.12)
**Carn.a.C18:2.OH**	0.02 (0.01, 0.04)
**Carn.a.C20:0**	0.03 (0.02, 0.05)
**Carn.a.C20:1**	0 (0, 0)
**Carn.a.C20:3**	0.06 (0.03, 0.1)
**Carn.a.C20:4**	0 (0, 0.01)

Values presented as medians (95% range) of neonatal metabolites in cord blood (µmol/L)

Supplemental Table S3. Associations of cord-blood individual metabolites and metabolite groups with SRS scores at age 6 and 13. Basic model.

	Differences in SRS score age 6 N = 716		Differences in SRS score age 13 N = 648	
Metabolite	P-value	Estimate (95%-Interval)	P-value	Estimate (95%-Interval)
Ala	0.92	-0.02 (-0.32 - 0.27)	0.51	0.25 (-0.05 - 0.55)
Arg	0.35	-0.21 (-0.5 - 0.08)	0.76	0.1 (-0.2 - 0.4)
Asn	0.97	-0.01 (-0.3 - 0.29)	0.45	0.3 (0 - 0.6)
Asp	0.42	0.19 (-0.1 - 0.48)	0.45	0.31 (0 - 0.62)
Cit	0.71	0.1 (-0.2 - 0.39)	0.54	0.22 (-0.07 - 0.52)
Gln	0.81	0.06 (-0.23 - 0.35)	0.72	0.14 (-0.17 - 0.45)
Glu	0.37	0.21 (-0.09 - 0.51)	0.51	0.28 (-0.02 - 0.58)
Gly	0.75	0.08 (-0.21 - 0.37)	0.51	0.26 (-0.04 - 0.57)
His	0.74	-0.08 (-0.38 - 0.21)	0.65	0.16 (-0.13 - 0.46)
Ile	0.56	0.13 (-0.16 - 0.43)	0.54	0.23 (-0.07 - 0.53)
Leu	0.82	0.05 (-0.24 - 0.34)	0.51	0.26 (-0.05 - 0.56)
Lys	0.60	0.12 (-0.17 - 0.41)	0.87	0.05 (-0.25 - 0.36)
Met	0.93	-0.02 (-0.31 - 0.28)	0.45	0.32 (0.01 - 0.62)
Orn	0.21	0.3 (0.01 - 0.6)	0.45	0.3 (0 - 0.6)
Phe	0.84	0.04 (-0.25 - 0.33)	0.62	0.19 (-0.12 - 0.49)
Pro	0.45	0.18 (-0.12 - 0.47)	0.51	0.27 (-0.03 - 0.58)
Trp	0.41	-0.19 (-0.48 - 0.1)	0.89	0.04 (-0.26 - 0.35)
Ser	0.30	0.25 (-0.05 - 0.54)	0.51	0.26 (-0.05 - 0.56)
Thr	0.82	-0.05 (-0.34 - 0.24)	0.51	0.27 (-0.03 - 0.56)
Tyr	0.82	0.05 (-0.24 - 0.35)	0.59	0.19 (-0.11 - 0.5)
Val	0.88	0.03 (-0.26 - 0.32)	0.51	0.26 (-0.04 - 0.57)
Cys	0.80	0.06 (-0.22 - 0.35)	0.89	0.04 (-0.26 - 0.35)
NEFA_14_0	0.08	-0.4 (-0.69 - -0.11)	0.87	0.05 (-0.25 - 0.35)
NEFA_15_0	0.06	-0.44 (-0.74 - -0.15)	0.96	-0.02 (-0.32 - 0.28)
NEFA_16_0	0.67	-0.1 (-0.39 - 0.19)	0.54	0.21 (-0.09 - 0.51)
NEFA_17_0	0.26	-0.26 (-0.55 - 0.03)	0.83	0.07 (-0.22 - 0.37)
NEFA_18_0	0.82	0.05 (-0.24 - 0.34)	0.51	0.27 (-0.03 - 0.57)
NEFA_24_0	0.60	-0.12 (-0.41 - 0.17)	0.54	0.21 (-0.09 - 0.5)
NEFA_26_0	0.33	-0.22 (-0.52 - 0.07)	0.65	0.16 (-0.13 - 0.46)
NEFA_14_1	0.06	-0.44 (-0.74 - -0.15)	0.88	-0.05 (-0.35 - 0.26)
NEFA_16_1	0.17	-0.33 (-0.62 - -0.04)	0.96	-0.01 (-0.32 - 0.29)
NEFA_17_1	0.10	-0.39 (-0.68 - -0.09)	0.96	-0.02 (-0.32 - 0.28)
NEFA_18_1	0.26	-0.27 (-0.56 - 0.02)	0.77	0.09 (-0.21 - 0.39)

NEFA_19_1	0.09	-0.39 (-0.68 - -0.1)	0.92	0.03 (-0.27 - 0.33)
NEFA_20_1	0.47	-0.17 (-0.46 - 0.13)	0.76	0.11 (-0.19 - 0.41)
NEFA_24_1	0.52	-0.15 (-0.44 - 0.14)	0.51	0.26 (-0.04 - 0.56)
NEFA_26_1	0.52	-0.15 (-0.44 - 0.14)	0.65	0.17 (-0.13 - 0.47)
NEFA_16_2	0.26	-0.26 (-0.56 - 0.03)	0.72	0.13 (-0.17 - 0.43)
NEFA_17_2	0.13	-0.36 (-0.65 - -0.07)	0.87	-0.05 (-0.35 - 0.25)
NEFA_18_2	0.41	-0.19 (-0.48 - 0.1)	0.65	0.17 (-0.13 - 0.46)
NEFA_18_3	0.23	-0.29 (-0.58 - 0.01)	0.76	0.1 (-0.2 - 0.39)
NEFA_20_2	0.72	-0.09 (-0.38 - 0.2)	0.55	0.2 (-0.1 - 0.5)
NEFA_20_3	0.55	-0.14 (-0.43 - 0.15)	0.77	0.09 (-0.21 - 0.38)
NEFA_20_4	0.27	-0.25 (-0.54 - 0.04)	0.96	0.02 (-0.27 - 0.32)
NEFA_20_5	0.08	-0.4 (-0.69 - -0.12)	0.58	-0.2 (-0.5 - 0.11)
NEFA_22_3	0.74	0.08 (-0.21 - 0.37)	0.54	0.2 (-0.09 - 0.5)
NEFA_22_4	0.47	-0.17 (-0.46 - 0.12)	0.85	0.06 (-0.24 - 0.36)
NEFA_22_5	0.45	-0.18 (-0.47 - 0.11)	1.00	0 (-0.3 - 0.3)
NEFA_22_6	0.27	-0.25 (-0.54 - 0.04)	0.92	0.03 (-0.27 - 0.33)
NEFA_24_2	0.81	-0.06 (-0.35 - 0.24)	0.40	0.36 (0.06 - 0.66)
NEFA_24_4	0.53	0.14 (-0.14 - 0.43)	0.51	0.28 (-0.02 - 0.58)
NEFA_24_5	0.89	-0.03 (-0.32 - 0.26)	0.54	0.21 (-0.09 - 0.51)
NEFA_26_2	0.98	0 (-0.29 - 0.28)	0.53	0.24 (-0.06 - 0.54)
PC.aa.C30.0	0.10	-0.39 (-0.68 - -0.09)	0.95	-0.03 (-0.33 - 0.28)
PC.aa.C32.0	0.30	-0.25 (-0.54 - 0.05)	0.73	-0.12 (-0.42 - 0.18)
PC.aa.C36.0	0.60	-0.12 (-0.41 - 0.17)	0.90	-0.04 (-0.35 - 0.27)
PC.aa.C38.0	0.30	-0.24 (-0.54 - 0.06)	0.72	-0.14 (-0.44 - 0.16)
PC.aa.C40.0	0.39	-0.2 (-0.49 - 0.09)	0.54	-0.22 (-0.51 - 0.08)
PC.aa.C42.0	0.26	-0.27 (-0.56 - 0.02)	0.54	-0.21 (-0.52 - 0.1)
PC.aa.C32.1	0.26	-0.27 (-0.57 - 0.03)	0.98	0.01 (-0.29 - 0.3)
PC.aa.C34.1	0.56	-0.14 (-0.43 - 0.16)	0.87	0.05 (-0.25 - 0.35)
PC.aa.C36.1	0.42	-0.19 (-0.48 - 0.11)	0.84	0.07 (-0.23 - 0.37)
PC.aa.C40.1	0.34	-0.22 (-0.51 - 0.07)	0.45	-0.3 (-0.6 - 0)
PC.aa.C30.3	0.26	-0.26 (-0.56 - 0.03)	0.76	-0.1 (-0.4 - 0.2)
PC.aa.C32.2	0.97	-0.01 (-0.3 - 0.28)	0.40	-0.36 (-0.67 - -0.06)
PC.aa.C32.3	0.74	-0.08 (-0.37 - 0.21)	0.72	0.13 (-0.17 - 0.42)
PC.aa.C34.2	0.60	-0.12 (-0.41 - 0.18)	0.76	0.11 (-0.19 - 0.41)
PC.aa.C34.3	0.27	-0.26 (-0.55 - 0.04)	0.90	0.04 (-0.26 - 0.33)
PC.aa.C34.4	0.31	-0.23 (-0.52 - 0.06)	0.96	0.02 (-0.28 - 0.32)
PC.aa.C34.5	0.06	-0.45 (-0.75 - -0.16)	0.83	-0.08 (-0.37 - 0.22)
PC.aa.C36.2	0.74	-0.08 (-0.38 - 0.21)	0.63	0.18 (-0.12 - 0.48)
PC.aa.C36.3	0.51	-0.15 (-0.45 - 0.14)	0.96	0.02 (-0.28 - 0.32)
PC.aa.C36.4	0.34	-0.22 (-0.51 - 0.07)	0.84	-0.06 (-0.37 - 0.24)
PC.aa.C36.5	0.07	-0.43 (-0.72 - -0.14)	0.76	-0.11 (-0.41 - 0.19)

PC.aa.C36.6	0.31	-0.23 (-0.52 - 0.06)	0.87	-0.05 (-0.35 - 0.25)
PC.aa.C38.2	0.30	-0.24 (-0.53 - 0.05)	0.70	-0.15 (-0.45 - 0.15)
PC.aa.C38.3	0.53	-0.15 (-0.44 - 0.15)	0.97	0.01 (-0.29 - 0.31)
PC.aa.C38.4	0.42	-0.19 (-0.48 - 0.1)	0.84	-0.07 (-0.38 - 0.24)
PC.aa.C38.5	0.47	-0.17 (-0.46 - 0.13)	0.84	-0.06 (-0.36 - 0.23)
PC.aa.C38.6	0.21	-0.29 (-0.58 - 0)	0.53	-0.24 (-0.55 - 0.06)
PC.aa.C40.2	0.28	-0.25 (-0.54 - 0.04)	0.40	-0.37 (-0.67 - -0.08)
PC.aa.C40.3	0.24	-0.28 (-0.57 - 0.01)	0.40	-0.33 (-0.63 - -0.04)
PC.aa.C40.4	0.72	-0.09 (-0.38 - 0.2)	0.76	-0.11 (-0.41 - 0.19)
PC.aa.C40.5	0.44	-0.18 (-0.47 - 0.11)	0.65	-0.17 (-0.47 - 0.13)
PC.aa.C40.6	0.30	-0.24 (-0.52 - 0.05)	0.62	-0.19 (-0.49 - 0.12)
PC.aa.C42.5	0.21	-0.29 (-0.58 - 0)	0.54	-0.22 (-0.52 - 0.08)
PC.aa.C43.6	0.82	-0.05 (-0.35 - 0.24)	0.96	0.01 (-0.29 - 0.32)
PC.aa.C44.12	0.21	-0.31 (-0.6 - -0.01)	0.72	-0.14 (-0.45 - 0.16)
PC.ae.C30.0	0.33	-0.22 (-0.51 - 0.07)	0.51	-0.26 (-0.56 - 0.04)
PC.ae.C32.0	0.42	-0.19 (-0.48 - 0.1)	0.65	-0.16 (-0.46 - 0.13)
PC.ae.C34.0	0.06	-0.49 (-0.78 - -0.19)	0.42	-0.32 (-0.62 - -0.02)
PC.ae.C36.0	0.81	-0.06 (-0.36 - 0.24)	0.87	-0.05 (-0.35 - 0.25)
PC.ae.C38.0	0.17	-0.33 (-0.62 - -0.04)	0.51	-0.27 (-0.57 - 0.03)
PC.ae.C40.0	0.18	-0.32 (-0.61 - -0.03)	0.96	-0.02 (-0.32 - 0.28)
PC.ae.C32.1	0.47	-0.17 (-0.46 - 0.13)	0.85	-0.06 (-0.36 - 0.24)
PC.ae.C34.1	0.21	-0.3 (-0.6 - -0.01)	0.76	-0.1 (-0.4 - 0.2)
PC.ae.C36.1	0.21	-0.29 (-0.59 - 0)	0.42	-0.32 (-0.62 - -0.02)
PC.ae.C40.1	0.82	-0.05 (-0.35 - 0.25)	0.83	-0.07 (-0.37 - 0.23)
PC.ae.C42.1	0.82	-0.05 (-0.34 - 0.24)	0.72	-0.13 (-0.44 - 0.18)
PC.ae.C32.2	0.06	-0.44 (-0.73 - -0.15)	0.42	-0.33 (-0.62 - -0.03)
PC.ae.C34.2	0.26	-0.26 (-0.56 - 0.03)	0.78	-0.09 (-0.39 - 0.21)
PC.ae.C34.3	0.20	-0.31 (-0.6 - -0.02)	0.76	-0.1 (-0.4 - 0.2)
PC.ae.C34.4	0.06	-0.44 (-0.73 - -0.16)	0.33	-0.46 (-0.76 - -0.16)
PC.ae.C36.2	0.13	-0.35 (-0.64 - -0.06)	0.53	-0.24 (-0.53 - 0.06)
PC.ae.C36.3	0.55	-0.14 (-0.43 - 0.15)	0.76	-0.1 (-0.39 - 0.2)
PC.ae.C36.4	0.30	-0.24 (-0.54 - 0.05)	0.54	-0.23 (-0.53 - 0.07)
PC.ae.C36.5	0.45	-0.18 (-0.47 - 0.12)	0.54	-0.23 (-0.53 - 0.08)
PC.ae.C38.2	0.10	-0.38 (-0.67 - -0.09)	0.51	-0.27 (-0.57 - 0.02)
PC.ae.C38.3	0.13	-0.36 (-0.65 - -0.07)	0.54	-0.22 (-0.52 - 0.08)
PC.ae.C38.4	0.31	-0.23 (-0.52 - 0.06)	0.72	-0.13 (-0.43 - 0.17)
PC.ae.C38.5	0.45	-0.18 (-0.48 - 0.12)	0.71	-0.15 (-0.45 - 0.16)
PC.ae.C38.6	0.30	-0.24 (-0.53 - 0.06)	0.52	-0.25 (-0.55 - 0.05)
PC.ae.C40.2	0.73	-0.08 (-0.37 - 0.21)	0.96	-0.02 (-0.32 - 0.28)
PC.ae.C40.3	0.15	-0.34 (-0.63 - -0.05)	0.78	-0.09 (-0.39 - 0.21)
PC.ae.C40.4	0.06	-0.43 (-0.72 - -0.14)	0.53	-0.24 (-0.53 - 0.06)

PC.ae.C40.5	0.15	-0.34 (-0.63 - -0.05)	0.40	-0.34 (-0.64 - -0.04)
PC.ae.C40.6	0.08	-0.41 (-0.7 - -0.13)	0.52	-0.25 (-0.55 - 0.05)
PC.ae.C42.3	0.47	-0.16 (-0.46 - 0.13)	0.72	-0.12 (-0.42 - 0.17)
PC.ae.C42.4	0.30	-0.24 (-0.52 - 0.05)	0.65	-0.16 (-0.46 - 0.13)
PC.ae.C42.5	0.52	-0.15 (-0.45 - 0.14)	0.72	-0.13 (-0.43 - 0.17)
PC.ae.C42.6	0.08	-0.4 (-0.69 - -0.11)	0.45	-0.31 (-0.61 - 0)
lyso.PC.a.C14.0	0.72	-0.09 (-0.38 - 0.2)	0.86	0.06 (-0.24 - 0.35)
lyso.PC.a.C16.0	0.47	0.17 (-0.13 - 0.47)	0.76	0.1 (-0.21 - 0.4)
lyso.PC.a.C18.0	0.53	0.15 (-0.15 - 0.44)	0.84	0.07 (-0.24 - 0.38)
lyso.PC.a.C16.1	0.92	-0.02 (-0.31 - 0.27)	0.76	0.11 (-0.19 - 0.41)
lyso.PC.a.C18.1	0.82	-0.05 (-0.35 - 0.25)	0.87	0.05 (-0.25 - 0.35)
lyso.PC.a.C18.2	0.97	-0.01 (-0.31 - 0.29)	0.51	0.26 (-0.04 - 0.57)
lyso.PC.a.C18.3	0.62	0.12 (-0.18 - 0.41)	0.53	0.23 (-0.06 - 0.53)
lyso.PC.a.C20.3	0.93	-0.02 (-0.31 - 0.28)	0.76	0.1 (-0.2 - 0.4)
lyso.PC.a.C20.4	0.82	-0.05 (-0.34 - 0.24)	0.76	0.1 (-0.2 - 0.41)
lyso.PC.a.C20.5	0.20	-0.31 (-0.6 - -0.02)	0.83	-0.07 (-0.37 - 0.23)
lyso.PC.a.C22.6	0.37	-0.21 (-0.5 - 0.09)	0.54	-0.21 (-0.51 - 0.1)
lyso.PC.e.C16.0	0.47	-0.16 (-0.45 - 0.12)	0.72	-0.13 (-0.43 - 0.16)
lyso.PC.e.C18.0	0.17	-0.33 (-0.61 - -0.04)	0.54	-0.21 (-0.51 - 0.08)
lyso.PC.e.C18.1	0.48	-0.16 (-0.46 - 0.13)	0.97	-0.01 (-0.3 - 0.28)
SM.a.C30.1	0.23	-0.28 (-0.57 - 0.01)	0.71	-0.14 (-0.44 - 0.16)
SM.a.C32.1	0.08	-0.4 (-0.69 - -0.11)	0.59	-0.19 (-0.48 - 0.11)
SM.a.C33.1	0.08	-0.4 (-0.69 - -0.11)	0.45	-0.3 (-0.59 - 0)
SM.a.C34.1	0.33	-0.23 (-0.52 - 0.07)	0.65	-0.17 (-0.47 - 0.13)
SM.a.C35.1	0.55	-0.14 (-0.43 - 0.15)	0.78	-0.09 (-0.39 - 0.21)
SM.a.C36.1	0.46	-0.17 (-0.46 - 0.12)	0.87	-0.05 (-0.35 - 0.25)
SM.a.C37.1	0.20	-0.31 (-0.61 - -0.02)	0.72	-0.12 (-0.42 - 0.17)
SM.a.C39.1	0.13	-0.36 (-0.64 - -0.07)	0.72	-0.13 (-0.43 - 0.17)
SM.a.C41.1	0.06	-0.44 (-0.73 - -0.15)	0.54	-0.23 (-0.53 - 0.07)
SM.a.C42.1	0.57	-0.13 (-0.42 - 0.16)	0.75	-0.12 (-0.42 - 0.18)
SM.a.C43.1	0.06	-0.43 (-0.72 - -0.15)	0.54	-0.22 (-0.51 - 0.08)
SM.a.C32.2	0.18	-0.33 (-0.62 - -0.03)	0.75	-0.12 (-0.43 - 0.19)
SM.a.C34.2	0.43	-0.18 (-0.47 - 0.11)	0.77	-0.09 (-0.39 - 0.21)
SM.a.C36.2	0.43	-0.18 (-0.48 - 0.11)	0.97	0.01 (-0.29 - 0.31)
SM.a.C36.3	0.74	-0.08 (-0.37 - 0.21)	0.84	-0.06 (-0.36 - 0.23)
SM.a.C38.2	0.47	-0.17 (-0.46 - 0.13)	0.78	-0.09 (-0.4 - 0.22)
SM.a.C38.3	0.72	-0.09 (-0.38 - 0.2)	0.99	0 (-0.31 - 0.3)
SM.a.C39.2	0.01	-0.6 (-0.9 - -0.31)	0.88	-0.05 (-0.34 - 0.25)
SM.a.C40.2	0.37	-0.21 (-0.5 - 0.09)	0.72	-0.13 (-0.44 - 0.18)
SM.a.C40.5	0.30	-0.24 (-0.53 - 0.05)	0.65	-0.18 (-0.49 - 0.13)
SM.a.C41.2	0.27	-0.26 (-0.55 - 0.03)	0.57	-0.19 (-0.49 - 0.1)

SM.a.C42.2	0.36	-0.21 (-0.51 - 0.08)	0.67	-0.16 (-0.46 - 0.14)
SM.a.C42.3	0.63	-0.11 (-0.4 - 0.18)	0.76	-0.1 (-0.4 - 0.2)
SM.a.C42.4	0.37	-0.21 (-0.5 - 0.09)	0.76	-0.1 (-0.4 - 0.2)
SM.a.C42.6	0.26	-0.27 (-0.56 - 0.02)	0.54	-0.22 (-0.53 - 0.08)
SM.a.C43.2	0.13	-0.35 (-0.64 - -0.07)	0.87	-0.05 (-0.36 - 0.25)
SM.a.C44.6	0.18	-0.32 (-0.61 - -0.03)	0.63	-0.18 (-0.48 - 0.13)
SM.e.C36.2	0.39	-0.2 (-0.49 - 0.09)	0.72	-0.12 (-0.42 - 0.18)
SM.e.C38.3	0.26	-0.26 (-0.55 - 0.03)	0.89	-0.04 (-0.34 - 0.26)
SM.e.C40.5	0.73	-0.09 (-0.38 - 0.21)	0.72	-0.13 (-0.43 - 0.18)
Carn.a.C2.0	0.54	0.14 (-0.15 - 0.43)	0.40	0.38 (0.08 - 0.67)
Carn.a.C3.0	0.81	0.06 (-0.23 - 0.35)	0.70	0.15 (-0.15 - 0.44)
Carn.a.C3.0.DC	0.86	-0.04 (-0.33 - 0.25)	0.76	0.11 (-0.19 - 0.41)
Carn.a.C4.0	0.76	-0.07 (-0.37 - 0.22)	0.72	0.14 (-0.16 - 0.44)
Carn.a.C5.0	0.56	0.14 (-0.16 - 0.43)	0.97	0.01 (-0.29 - 0.32)
Carn.a.C6.0	0.79	0.07 (-0.23 - 0.36)	0.40	0.34 (0.04 - 0.64)
Carn.a.C6.0.OH	0.77	0.07 (-0.22 - 0.36)	0.76	0.1 (-0.2 - 0.4)
Carn.a.C8.0	0.71	-0.1 (-0.39 - 0.2)	0.83	0.07 (-0.22 - 0.37)
Carn.a.C8.1	0.81	-0.06 (-0.35 - 0.23)	0.76	-0.11 (-0.41 - 0.19)
Carn.a.C9.0	0.81	0.06 (-0.23 - 0.36)	0.72	0.14 (-0.17 - 0.44)
Carn.a.C10.0	0.42	-0.19 (-0.48 - 0.1)	0.82	0.08 (-0.22 - 0.38)
Carn.a.C10.1	0.72	0.09 (-0.2 - 0.38)	0.54	0.21 (-0.09 - 0.51)
Carn.a.C12.0	0.92	-0.02 (-0.31 - 0.27)	0.76	0.11 (-0.19 - 0.42)
Carn.a.C14.1	0.88	-0.03 (-0.32 - 0.26)	0.83	0.08 (-0.23 - 0.38)
Carn.a.C14.2	0.57	0.13 (-0.16 - 0.42)	0.54	0.24 (-0.07 - 0.54)
Carn.a.C15.0	0.81	0.06 (-0.23 - 0.35)	0.59	0.19 (-0.11 - 0.5)
Carn.a.C16.0	0.26	0.27 (-0.02 - 0.57)	0.83	0.07 (-0.23 - 0.38)
Carn.a.C16.0.Oxo	0.87	0.03 (-0.26 - 0.33)	0.97	0.01 (-0.29 - 0.31)
Carn.a.C16.1	0.47	0.17 (-0.13 - 0.46)	0.54	0.21 (-0.1 - 0.51)
Carn.a.C16.2	0.73	0.09 (-0.2 - 0.38)	0.96	0.02 (-0.29 - 0.33)
Carn.a.C18.0	0.74	0.08 (-0.21 - 0.37)	0.78	0.09 (-0.21 - 0.38)
Carn.a.C18.1	0.48	0.16 (-0.13 - 0.45)	0.96	0.02 (-0.29 - 0.32)
Carn.a.C18.2	0.06	0.48 (0.19 - 0.77)	0.51	0.28 (-0.02 - 0.59)
Carn.a.C18.2.OH	0.75	0.08 (-0.22 - 0.37)	0.96	0.02 (-0.29 - 0.32)
Carn.a.C20.0	0.82	0.05 (-0.24 - 0.34)	0.76	0.1 (-0.2 - 0.4)
Carn.a.C20.1	0.61	0.12 (-0.18 - 0.41)	0.59	0.19 (-0.11 - 0.49)
Carn.a.C20.3	0.57	0.13 (-0.16 - 0.42)	0.84	0.07 (-0.24 - 0.37)
Carn.a.C20.4	0.69	0.1 (-0.19 - 0.39)	0.59	0.19 (-0.11 - 0.5)
BCAA	0.81	0.06 (-0.23 - 0.35)	0.51	0.27 (-0.03 - 0.58)
AAA	0.86	-0.04 (-0.33 - 0.26)	0.69	0.16 (-0.15 - 0.46)
EAA	0.88	0.03 (-0.26 - 0.32)	0.51	0.26 (-0.04 - 0.56)
NEAA	0.59	0.12 (-0.17 - 0.42)	0.40	0.34 (0.04 - 0.65)

AA	0.73	0.09 (-0.21 - 0.38)	0.42	0.32 (0.02 - 0.63)
SATURATEDNEFA	0.60	-0.12 (-0.41 - 0.17)	0.54	0.21 (-0.09 - 0.51)
MONOUNSATNEFA	0.21	-0.3 (-0.59 - 0)	0.84	0.06 (-0.24 - 0.36)
POLYUNSATNEFA	0.33	-0.23 (-0.52 - 0.06)	0.72	0.14 (-0.16 - 0.43)
NEFA	0.37	-0.21 (-0.5 - 0.09)	0.70	0.15 (-0.15 - 0.45)
SATURATEDPCaa	0.21	-0.3 (-0.59 - 0)	0.72	-0.13 (-0.43 - 0.18)
MONOUNSATPCaa	0.48	-0.16 (-0.46 - 0.13)	0.87	0.05 (-0.25 - 0.35)
POLYUNSATPCaa	0.33	-0.22 (-0.52 - 0.07)	0.87	-0.05 (-0.35 - 0.25)
PCaa	0.34	-0.22 (-0.51 - 0.07)	0.91	-0.04 (-0.34 - 0.27)
SATURATEDPCae	0.14	-0.35 (-0.64 - -0.06)	0.75	-0.12 (-0.42 - 0.18)
MONOUNSATPCae	0.26	-0.27 (-0.57 - 0.02)	0.66	-0.16 (-0.46 - 0.14)
POLYUNSATPCae	0.21	-0.3 (-0.59 - 0)	0.54	-0.23 (-0.53 - 0.07)
PCae	0.20	-0.31 (-0.61 - -0.02)	0.54	-0.21 (-0.51 - 0.09)
SATURATEDLYSOPCa	0.50	0.16 (-0.14 - 0.45)	0.77	0.09 (-0.21 - 0.4)
MONOUNSATLYSOPCa	0.86	-0.04 (-0.33 - 0.26)	0.84	0.07 (-0.23 - 0.37)
POLYUNSATLYSOPCa	0.82	-0.05 (-0.34 - 0.25)	0.70	0.15 (-0.15 - 0.46)
LYSOPCa	0.74	0.08 (-0.21 - 0.38)	0.76	0.11 (-0.19 - 0.42)
SATURATEDLYSOPCe	0.21	-0.3 (-0.58 - -0.01)	0.54	-0.21 (-0.5 - 0.09)
lyso.PC.e.C18.1	0.48	-0.16 (-0.46 - 0.13)	0.97	-0.01 (-0.3 - 0.28)
LYSOPCe	0.21	-0.29 (-0.58 - 0)	0.59	-0.18 (-0.48 - 0.11)
MONOUNSATSM	0.30	-0.24 (-0.54 - 0.05)	0.69	-0.15 (-0.45 - 0.14)
POLYUNSATSM	0.34	-0.22 (-0.51 - 0.07)	0.72	-0.13 (-0.43 - 0.18)
SM	0.31	-0.23 (-0.53 - 0.06)	0.71	-0.14 (-0.44 - 0.16)
Carn	0.26	0.26 (-0.03 - 0.56)	0.40	0.38 (0.1 - 0.67)
SHORTCHAINCARNa	0.57	0.13 (-0.16 - 0.42)	0.40	0.35 (0.06 - 0.65)
MEDIUMCHAINCARNa	0.87	-0.03 (-0.33 - 0.26)	0.71	0.14 (-0.16 - 0.45)
LONGCHAINCARNa	0.39	0.2 (-0.09 - 0.5)	0.72	0.14 (-0.16 - 0.45)
ACYLCARN	0.56	0.13 (-0.16 - 0.43)	0.40	0.33 (0.04 - 0.63)

Values represent absolute differences in SRS score and corresponding p-values from linear regression models that reflect the difference in SRS score at age 6 and 13 per SDS increase in cord-blood metabolite concentrations (µmol/L). Model includes sex and age at outcome. AA amino acids, NEFA non-esterified fatty acids, PC.aa diacyl-phosphatidylcholines, PC.ae acyl-alkyl-phosphatidylcholines, lyso.PC.a. acyl-lysophosphatidylcholines, lyso.PC.e alkyl-lysophosphatidylcholines, Carn.a acylcarnitines, SM sphingomyelines

Supplemental Table S4. Associations of cord-blood individual metabolites and metabolite groups with SRS scores at age 6 and 13. Main model.

	Differences in SRS score age 6 N = 716		Differences in SRS score age 13 N = 648	
Metabolite	P-value	Estimate (95%-Interval)	P-value	Estimate (95%-Interval)
Ala	1.00	-0.01 (-0.3 - 0.29)	0.75	0.25 (-0.05 - 0.55)
Arg	0.45	-0.23 (-0.52 - 0.06)	0.99	0.03 (-0.28 - 0.33)
Asn	0.88	0.03 (-0.26 - 0.32)	0.75	0.27 (-0.02 - 0.57)
Asp	0.44	0.24 (-0.05 - 0.53)	0.75	0.28 (-0.02 - 0.59)
Cit	0.62	0.13 (-0.16 - 0.43)	0.82	0.18 (-0.12 - 0.48)
Gln	0.81	0.07 (-0.22 - 0.36)	0.89	0.13 (-0.17 - 0.44)
Glu	0.50	0.2 (-0.09 - 0.49)	0.82	0.21 (-0.09 - 0.51)
Gly	0.62	0.13 (-0.16 - 0.42)	0.82	0.2 (-0.1 - 0.5)
His	0.77	-0.08 (-0.37 - 0.22)	0.89	0.1 (-0.2 - 0.39)
Ile	0.77	0.08 (-0.22 - 0.37)	0.82	0.19 (-0.11 - 0.49)
Leu	0.86	0.04 (-0.25 - 0.33)	0.82	0.21 (-0.1 - 0.51)
Lys	0.88	0.03 (-0.26 - 0.32)	0.99	-0.02 (-0.33 - 0.29)
Met	0.98	0 (-0.29 - 0.29)	0.75	0.26 (-0.04 - 0.57)
Orn	0.33	0.31 (0.02 - 0.6)	0.75	0.25 (-0.05 - 0.56)
Phe	0.91	0.03 (-0.26 - 0.32)	0.89	0.12 (-0.18 - 0.43)
Pro	0.54	0.17 (-0.12 - 0.45)	0.82	0.2 (-0.1 - 0.51)
Trp	0.62	-0.13 (-0.42 - 0.16)	0.98	0.04 (-0.26 - 0.34)
Ser	0.45	0.24 (-0.05 - 0.53)	0.82	0.2 (-0.1 - 0.51)
Thr	0.86	-0.05 (-0.34 - 0.24)	0.82	0.23 (-0.07 - 0.53)
Tyr	0.97	0.01 (-0.28 - 0.31)	0.89	0.13 (-0.18 - 0.44)
Val	0.97	0.01 (-0.28 - 0.31)	0.82	0.2 (-0.11 - 0.5)
Cys	0.97	0.01 (-0.28 - 0.3)	1.00	0 (-0.31 - 0.3)
NEFA_14_0	0.24	-0.36 (-0.64 - -0.07)	0.94	0.07 (-0.23 - 0.36)
NEFA_15_0	0.18	-0.39 (-0.68 - -0.1)	1.00	0 (-0.29 - 0.3)
NEFA_16_0	0.76	-0.08 (-0.37 - 0.21)	0.82	0.23 (-0.08 - 0.53)
NEFA_17_0	0.52	-0.19 (-0.48 - 0.1)	0.89	0.14 (-0.16 - 0.44)
NEFA_18_0	0.82	0.06 (-0.23 - 0.35)	0.75	0.29 (-0.01 - 0.59)
NEFA_24_0	0.75	-0.09 (-0.37 - 0.2)	0.75	0.25 (-0.04 - 0.55)
NEFA_26_0	0.45	-0.23 (-0.53 - 0.06)	0.82	0.18 (-0.12 - 0.48)
NEFA_14_1	0.18	-0.4 (-0.69 - -0.1)	0.99	-0.01 (-0.32 - 0.29)
NEFA_16_1	0.38	-0.29 (-0.58 - 0)	0.99	0.02 (-0.28 - 0.33)
NEFA_17_1	0.28	-0.33 (-0.62 - -0.04)	0.99	0.01 (-0.29 - 0.31)
NEFA_18_1	0.41	-0.26 (-0.55 - 0.03)	0.89	0.09 (-0.21 - 0.39)
NEFA_19_1	0.24	-0.36 (-0.65 - -0.07)	0.94	0.07 (-0.23 - 0.37)
NEFA_20_1	0.62	-0.14 (-0.43 - 0.15)	0.89	0.11 (-0.19 - 0.41)

NEFA_24_1	0.62	-0.13 (-0.41 - 0.16)	0.82	0.23 (-0.06 - 0.53)
NEFA_26_1	0.52	-0.19 (-0.48 - 0.1)	0.87	0.17 (-0.13 - 0.47)
NEFA_16_2	0.42	-0.26 (-0.55 - 0.03)	0.89	0.12 (-0.18 - 0.42)
NEFA_17_2	0.36	-0.3 (-0.59 - -0.01)	0.95	-0.05 (-0.35 - 0.25)
NEFA_18_2	0.53	-0.18 (-0.47 - 0.11)	0.87	0.16 (-0.14 - 0.46)
NEFA_18_3	0.38	-0.28 (-0.57 - 0.01)	0.89	0.1 (-0.2 - 0.39)
NEFA_20_2	0.84	-0.05 (-0.35 - 0.24)	0.82	0.2 (-0.1 - 0.49)
NEFA_20_3	0.64	-0.12 (-0.4 - 0.17)	0.93	0.07 (-0.22 - 0.36)
NEFA_20_4	0.43	-0.24 (-0.53 - 0.04)	0.99	0.02 (-0.28 - 0.31)
NEFA_20_5	0.28	-0.34 (-0.63 - -0.05)	0.82	-0.21 (-0.52 - 0.09)
NEFA_22_3	0.97	0.01 (-0.28 - 0.3)	0.82	0.18 (-0.11 - 0.47)
NEFA_22_4	0.58	-0.15 (-0.44 - 0.14)	0.89	0.11 (-0.2 - 0.41)
NEFA_22_5	0.62	-0.13 (-0.42 - 0.16)	0.99	0.03 (-0.27 - 0.33)
NEFA_22_6	0.53	-0.17 (-0.47 - 0.12)	0.95	0.05 (-0.25 - 0.35)
NEFA_24_2	0.81	-0.06 (-0.35 - 0.23)	0.69	0.35 (0.06 - 0.65)
NEFA_24_4	0.62	0.14 (-0.15 - 0.43)	0.75	0.29 (-0.01 - 0.59)
NEFA_24_5	0.96	-0.01 (-0.3 - 0.27)	0.82	0.21 (-0.09 - 0.51)
NEFA_26_2	0.85	-0.05 (-0.33 - 0.24)	0.82	0.23 (-0.07 - 0.54)
PC.aa.C30.0	0.18	-0.41 (-0.71 - -0.11)	0.94	-0.06 (-0.37 - 0.25)
PC.aa.C32.0	0.40	-0.28 (-0.58 - 0.02)	0.87	-0.16 (-0.47 - 0.15)
PC.aa.C36.0	0.83	-0.06 (-0.34 - 0.23)	0.99	0.01 (-0.3 - 0.31)
PC.aa.C38.0	0.67	-0.12 (-0.42 - 0.18)	0.89	-0.1 (-0.4 - 0.2)
PC.aa.C40.0	0.75	-0.09 (-0.38 - 0.2)	0.89	-0.14 (-0.44 - 0.16)
PC.aa.C42.0	0.62	-0.14 (-0.44 - 0.16)	0.89	-0.12 (-0.44 - 0.2)
PC.aa.C32.1	0.33	-0.33 (-0.63 - -0.03)	0.98	-0.04 (-0.34 - 0.26)
PC.aa.C34.1	0.57	-0.16 (-0.46 - 0.14)	0.99	0.01 (-0.29 - 0.31)
PC.aa.C36.1	0.48	-0.21 (-0.51 - 0.08)	0.99	0.03 (-0.27 - 0.34)
PC.aa.C40.1	0.67	-0.12 (-0.41 - 0.17)	0.82	-0.24 (-0.54 - 0.06)
PC.aa.C30.3	0.38	-0.28 (-0.57 - 0.01)	0.89	-0.09 (-0.39 - 0.2)
PC.aa.C32.2	0.86	-0.05 (-0.34 - 0.24)	0.69	-0.39 (-0.69 - -0.09)
PC.aa.C32.3	0.75	-0.09 (-0.38 - 0.19)	0.82	0.18 (-0.12 - 0.48)
PC.aa.C34.2	0.70	-0.11 (-0.41 - 0.18)	0.89	0.09 (-0.21 - 0.39)
PC.aa.C34.3	0.43	-0.26 (-0.55 - 0.04)	0.99	0.03 (-0.27 - 0.33)
PC.aa.C34.4	0.45	-0.23 (-0.52 - 0.06)	0.94	0.07 (-0.23 - 0.37)
PC.aa.C34.5	0.14	-0.46 (-0.75 - -0.16)	0.89	-0.11 (-0.4 - 0.19)
PC.aa.C36.2	0.77	-0.08 (-0.37 - 0.22)	0.89	0.14 (-0.16 - 0.45)
PC.aa.C36.3	0.55	-0.16 (-0.46 - 0.13)	0.99	-0.03 (-0.33 - 0.28)
PC.aa.C36.4	0.50	-0.21 (-0.5 - 0.09)	0.95	-0.05 (-0.36 - 0.25)
PC.aa.C36.5	0.24	-0.37 (-0.67 - -0.07)	0.89	-0.1 (-0.41 - 0.21)
PC.aa.C36.6	0.57	-0.16 (-0.45 - 0.13)	0.99	-0.01 (-0.31 - 0.29)
PC.aa.C38.2	0.49	-0.21 (-0.5 - 0.08)	0.89	-0.13 (-0.43 - 0.16)

PC.aa.C38.3	0.68	-0.11 (-0.4 - 0.18)	0.99	0.03 (-0.28 - 0.33)
PC.aa.C38.4	0.58	-0.15 (-0.43 - 0.14)	0.99	-0.02 (-0.33 - 0.28)
PC.aa.C38.5	0.67	-0.12 (-0.41 - 0.18)	0.99	-0.01 (-0.31 - 0.29)
PC.aa.C38.6	0.62	-0.14 (-0.44 - 0.16)	0.89	-0.11 (-0.42 - 0.21)
PC.aa.C40.2	0.53	-0.18 (-0.47 - 0.11)	0.69	-0.32 (-0.62 - -0.03)
PC.aa.C40.3	0.45	-0.22 (-0.51 - 0.07)	0.69	-0.33 (-0.62 - -0.03)
PC.aa.C40.4	0.98	0.01 (-0.29 - 0.3)	0.99	0.01 (-0.3 - 0.32)
PC.aa.C40.5	0.74	-0.1 (-0.4 - 0.2)	0.94	-0.06 (-0.37 - 0.25)
PC.aa.C40.6	0.86	-0.05 (-0.36 - 0.26)	0.99	-0.01 (-0.34 - 0.31)
PC.aa.C42.5	0.62	-0.13 (-0.43 - 0.16)	0.89	-0.14 (-0.45 - 0.17)
PC.aa.C43.6	0.78	0.07 (-0.23 - 0.36)	0.89	0.12 (-0.19 - 0.43)
PC.aa.C44.12	0.50	-0.22 (-0.52 - 0.08)	0.94	-0.06 (-0.37 - 0.25)
PC.ae.C30.0	0.49	-0.22 (-0.51 - 0.07)	0.75	-0.28 (-0.58 - 0.02)
PC.ae.C32.0	0.52	-0.19 (-0.49 - 0.1)	0.82	-0.19 (-0.49 - 0.11)
PC.ae.C34.0	0.18	-0.4 (-0.7 - -0.1)	0.82	-0.21 (-0.52 - 0.09)
PC.ae.C36.0	0.99	0 (-0.3 - 0.29)	0.99	-0.01 (-0.31 - 0.29)
PC.ae.C38.0	0.45	-0.23 (-0.52 - 0.06)	0.87	-0.17 (-0.47 - 0.13)
PC.ae.C40.0	0.45	-0.22 (-0.51 - 0.07)	0.99	0.03 (-0.27 - 0.34)
PC.ae.C32.1	0.52	-0.19 (-0.49 - 0.11)	0.95	-0.05 (-0.36 - 0.25)
PC.ae.C34.1	0.33	-0.32 (-0.62 - -0.02)	0.89	-0.13 (-0.43 - 0.17)
PC.ae.C36.1	0.50	-0.2 (-0.5 - 0.09)	0.75	-0.25 (-0.56 - 0.05)
PC.ae.C40.1	0.75	-0.09 (-0.39 - 0.21)	0.89	-0.1 (-0.4 - 0.2)
PC.ae.C42.1	0.99	0 (-0.29 - 0.29)	0.89	-0.1 (-0.41 - 0.21)
PC.ae.C32.2	0.14	-0.44 (-0.74 - -0.15)	0.69	-0.32 (-0.62 - -0.01)
PC.ae.C34.2	0.45	-0.23 (-0.52 - 0.06)	0.89	-0.1 (-0.4 - 0.2)
PC.ae.C34.3	0.45	-0.25 (-0.54 - 0.05)	0.99	-0.03 (-0.33 - 0.27)
PC.ae.C34.4	0.15	-0.42 (-0.71 - -0.14)	0.69	-0.44 (-0.74 - -0.15)
PC.ae.C36.2	0.45	-0.24 (-0.53 - 0.05)	0.82	-0.18 (-0.48 - 0.12)
PC.ae.C36.3	0.77	-0.08 (-0.37 - 0.21)	0.94	-0.06 (-0.35 - 0.24)
PC.ae.C36.4	0.50	-0.21 (-0.5 - 0.09)	0.82	-0.19 (-0.49 - 0.11)
PC.ae.C36.5	0.62	-0.13 (-0.42 - 0.17)	0.82	-0.18 (-0.48 - 0.12)
PC.ae.C38.2	0.28	-0.34 (-0.63 - -0.04)	0.82	-0.21 (-0.51 - 0.08)
PC.ae.C38.3	0.40	-0.27 (-0.56 - 0.03)	0.87	-0.16 (-0.46 - 0.14)
PC.ae.C38.4	0.57	-0.16 (-0.45 - 0.13)	0.94	-0.07 (-0.37 - 0.23)
PC.ae.C38.5	0.62	-0.13 (-0.43 - 0.16)	0.91	-0.08 (-0.38 - 0.22)
PC.ae.C38.6	0.58	-0.15 (-0.45 - 0.14)	0.82	-0.2 (-0.5 - 0.1)
PC.ae.C40.2	0.82	-0.06 (-0.35 - 0.23)	0.99	-0.02 (-0.31 - 0.28)
PC.ae.C40.3	0.38	-0.28 (-0.58 - 0.01)	0.98	-0.04 (-0.35 - 0.27)
PC.ae.C40.4	0.28	-0.34 (-0.65 - -0.04)	0.89	-0.14 (-0.44 - 0.17)
PC.ae.C40.5	0.38	-0.29 (-0.58 - 0.01)	0.75	-0.26 (-0.56 - 0.05)
PC.ae.C40.6	0.45	-0.25 (-0.56 - 0.05)	0.89	-0.1 (-0.41 - 0.22)

PC.ae.C42.3	0.71	-0.11 (-0.4 - 0.18)	0.89	-0.1 (-0.4 - 0.19)
PC.ae.C42.4	0.57	-0.15 (-0.44 - 0.14)	0.89	-0.09 (-0.39 - 0.21)
PC.ae.C42.5	0.75	-0.09 (-0.39 - 0.21)	0.99	-0.03 (-0.34 - 0.27)
PC.ae.C42.6	0.45	-0.25 (-0.56 - 0.05)	0.87	-0.17 (-0.48 - 0.15)
lyso.PC.a.C14.0	0.71	-0.11 (-0.4 - 0.18)	0.98	0.04 (-0.26 - 0.34)
lyso.PC.a.C16.0	0.53	0.18 (-0.12 - 0.47)	0.91	0.08 (-0.22 - 0.39)
lyso.PC.a.C18.0	0.53	0.18 (-0.11 - 0.47)	0.94	0.07 (-0.23 - 0.38)
lyso.PC.a.C16.1	0.62	-0.14 (-0.44 - 0.16)	0.94	0.06 (-0.25 - 0.37)
lyso.PC.a.C18.1	0.53	-0.18 (-0.48 - 0.13)	0.94	-0.07 (-0.38 - 0.24)
lyso.PC.a.C18.2	0.76	-0.09 (-0.39 - 0.22)	0.82	0.21 (-0.1 - 0.52)
lyso.PC.a.C18.3	0.89	0.04 (-0.26 - 0.33)	0.82	0.2 (-0.09 - 0.5)
lyso.PC.a.C20.3	0.70	-0.11 (-0.41 - 0.19)	0.97	0.04 (-0.27 - 0.36)
lyso.PC.a.C20.4	0.57	-0.16 (-0.45 - 0.14)	0.94	0.07 (-0.24 - 0.38)
lyso.PC.a.C20.5	0.40	-0.28 (-0.58 - 0.02)	0.94	-0.07 (-0.37 - 0.24)
lyso.PC.a.C22.6	0.55	-0.17 (-0.46 - 0.13)	0.87	-0.17 (-0.47 - 0.14)
lyso.PC.e.C16.0	0.60	-0.14 (-0.43 - 0.14)	0.89	-0.1 (-0.4 - 0.19)
lyso.PC.e.C18.0	0.45	-0.24 (-0.53 - 0.06)	0.89	-0.13 (-0.43 - 0.18)
lyso.PC.e.C18.1	0.57	-0.15 (-0.44 - 0.15)	0.99	-0.01 (-0.3 - 0.29)
SM.a.C30.1	0.43	-0.25 (-0.54 - 0.04)	0.89	-0.13 (-0.43 - 0.17)
SM.a.C32.1	0.28	-0.34 (-0.63 - -0.04)	0.89	-0.14 (-0.44 - 0.16)
SM.a.C33.1	0.30	-0.32 (-0.61 - -0.03)	0.75	-0.25 (-0.55 - 0.05)
SM.a.C34.1	0.53	-0.18 (-0.48 - 0.11)	0.89	-0.13 (-0.43 - 0.17)
SM.a.C35.1	0.82	-0.06 (-0.35 - 0.23)	0.99	-0.02 (-0.32 - 0.28)
SM.a.C36.1	0.53	-0.18 (-0.47 - 0.11)	0.94	-0.06 (-0.36 - 0.24)
SM.a.C37.1	0.44	-0.26 (-0.55 - 0.04)	0.95	-0.05 (-0.34 - 0.25)
SM.a.C39.1	0.36	-0.3 (-0.59 - -0.01)	0.95	-0.05 (-0.35 - 0.25)
SM.a.C41.1	0.18	-0.4 (-0.69 - -0.11)	0.87	-0.16 (-0.46 - 0.15)
SM.a.C42.1	0.76	-0.08 (-0.37 - 0.21)	0.89	-0.1 (-0.4 - 0.19)
SM.a.C43.1	0.28	-0.34 (-0.64 - -0.05)	0.87	-0.16 (-0.46 - 0.14)
SM.a.C32.2	0.36	-0.29 (-0.58 - 0)	0.91	-0.08 (-0.39 - 0.22)
SM.a.C34.2	0.54	-0.17 (-0.46 - 0.12)	0.93	-0.07 (-0.37 - 0.23)
SM.a.C36.2	0.52	-0.19 (-0.48 - 0.1)	0.99	0.03 (-0.28 - 0.33)
SM.a.C36.3	0.75	-0.09 (-0.38 - 0.2)	0.99	-0.02 (-0.32 - 0.28)
SM.a.C38.2	0.62	-0.13 (-0.43 - 0.16)	0.91	-0.09 (-0.4 - 0.22)
SM.a.C38.3	0.77	-0.08 (-0.37 - 0.22)	1.00	0 (-0.31 - 0.31)
SM.a.C39.2	0.08	-0.54 (-0.83 - -0.24)	0.99	0.02 (-0.28 - 0.32)
SM.a.C40.2	0.53	-0.18 (-0.47 - 0.11)	0.89	-0.13 (-0.44 - 0.18)
SM.a.C40.5	0.50	-0.2 (-0.49 - 0.1)	0.82	-0.19 (-0.5 - 0.13)
SM.a.C41.2	0.50	-0.19 (-0.49 - 0.1)	0.89	-0.13 (-0.42 - 0.17)
SM.a.C42.2	0.48	-0.22 (-0.51 - 0.07)	0.82	-0.2 (-0.5 - 0.1)
SM.a.C42.3	0.76	-0.08 (-0.37 - 0.21)	0.89	-0.1 (-0.41 - 0.2)

SM.a.C42.4	0.53	-0.18 (-0.47 - 0.12)	0.98	-0.04 (-0.34 - 0.26)
SM.a.C42.6	0.54	-0.17 (-0.47 - 0.13)	0.89	-0.11 (-0.42 - 0.2)
SM.a.C43.2	0.33	-0.31 (-0.59 - -0.02)	0.99	-0.02 (-0.32 - 0.28)
SM.a.C44.6	0.52	-0.2 (-0.49 - 0.1)	0.94	-0.06 (-0.38 - 0.25)
SM.e.C36.2	0.62	-0.14 (-0.43 - 0.16)	0.92	-0.08 (-0.38 - 0.22)
SM.e.C38.3	0.49	-0.2 (-0.5 - 0.09)	0.99	-0.03 (-0.32 - 0.27)
SM.e.C40.5	0.76	-0.08 (-0.37 - 0.21)	0.89	-0.11 (-0.41 - 0.19)
Carn.a.C2.0	0.64	0.12 (-0.17 - 0.41)	0.69	0.37 (0.08 - 0.66)
Carn.a.C3.0	0.97	0.02 (-0.28 - 0.31)	0.89	0.14 (-0.16 - 0.44)
Carn.a.C3.0.DC	0.70	-0.11 (-0.4 - 0.18)	0.91	0.08 (-0.22 - 0.38)
Carn.a.C4.0	0.75	-0.09 (-0.39 - 0.2)	0.89	0.1 (-0.21 - 0.4)
Carn.a.C5.0	0.97	0.01 (-0.29 - 0.31)	0.94	-0.06 (-0.37 - 0.25)
Carn.a.C6.0	0.97	-0.02 (-0.32 - 0.28)	0.75	0.26 (-0.05 - 0.57)
Carn.a.C6.0.OH	0.79	0.07 (-0.22 - 0.35)	0.95	0.05 (-0.25 - 0.35)
Carn.a.C8.0	0.73	-0.1 (-0.39 - 0.2)	0.94	0.06 (-0.23 - 0.36)
Carn.a.C8.1	0.62	-0.14 (-0.44 - 0.15)	0.87	-0.17 (-0.47 - 0.14)
Carn.a.C9.0	0.77	0.08 (-0.21 - 0.37)	0.89	0.13 (-0.17 - 0.44)
Carn.a.C10.0	0.53	-0.18 (-0.47 - 0.11)	0.95	0.05 (-0.25 - 0.36)
Carn.a.C10.1	0.77	0.07 (-0.22 - 0.36)	0.82	0.19 (-0.12 - 0.5)
Carn.a.C12.0	0.91	-0.03 (-0.32 - 0.26)	0.89	0.12 (-0.19 - 0.42)
Carn.a.C14.1	0.86	-0.04 (-0.33 - 0.25)	0.94	0.06 (-0.25 - 0.36)
Carn.a.C14.2	0.71	0.1 (-0.19 - 0.39)	0.82	0.2 (-0.11 - 0.5)
Carn.a.C15.0	0.85	0.05 (-0.24 - 0.34)	0.82	0.19 (-0.11 - 0.49)
Carn.a.C16.0	0.36	0.3 (0.01 - 0.6)	0.89	0.1 (-0.2 - 0.4)
Carn.a.C16.0.Oxo	0.88	0.04 (-0.25 - 0.33)	1.00	0 (-0.3 - 0.3)
Carn.a.C16.1	0.58	0.15 (-0.15 - 0.44)	0.82	0.2 (-0.1 - 0.5)
Carn.a.C16.2	0.78	0.07 (-0.22 - 0.36)	0.99	0.02 (-0.29 - 0.32)
Carn.a.C18.0	0.86	0.04 (-0.25 - 0.33)	0.89	0.09 (-0.21 - 0.38)
Carn.a.C18.1	0.53	0.17 (-0.12 - 0.47)	0.99	0.01 (-0.29 - 0.31)
Carn.a.C18.2	0.08	0.51 (0.22 - 0.8)	0.69	0.31 (0.01 - 0.62)
Carn.a.C18.2.OH	0.77	0.08 (-0.21 - 0.37)	1.00	0 (-0.31 - 0.31)
Carn.a.C20.0	0.86	0.05 (-0.24 - 0.33)	0.89	0.09 (-0.2 - 0.39)
Carn.a.C20.1	0.74	0.1 (-0.19 - 0.39)	0.82	0.18 (-0.11 - 0.48)
Carn.a.C20.3	0.68	0.12 (-0.17 - 0.41)	0.95	0.05 (-0.25 - 0.35)
Carn.a.C20.4	0.75	0.09 (-0.19 - 0.38)	0.87	0.17 (-0.13 - 0.48)
BCAA	0.89	0.04 (-0.26 - 0.33)	0.82	0.21 (-0.09 - 0.52)
AAA	0.88	-0.03 (-0.33 - 0.26)	0.89	0.11 (-0.2 - 0.41)
EAA	1.00	0 (-0.29 - 0.29)	0.82	0.18 (-0.12 - 0.49)
NEAA	0.62	0.14 (-0.16 - 0.43)	0.75	0.29 (-0.02 - 0.59)
AA	0.76	0.08 (-0.21 - 0.37)	0.75	0.26 (-0.05 - 0.56)
SATURATEDNEFA	0.74	-0.1 (-0.39 - 0.2)	0.82	0.23 (-0.07 - 0.53)

MONOUNSATNEFA	0.38	-0.28 (-0.57 - 0.01)	0.94	0.07 (-0.23 - 0.38)
POLYUNSATNEFA	0.49	-0.21 (-0.5 - 0.08)	0.89	0.13 (-0.17 - 0.43)
NEFA	0.52	-0.19 (-0.48 - 0.11)	0.87	0.16 (-0.14 - 0.46)
SATURATEDPCaa	0.38	-0.29 (-0.59 - 0.01)	0.89	-0.14 (-0.45 - 0.16)
MONOUNSATPCaa	0.52	-0.19 (-0.49 - 0.11)	0.99	0.01 (-0.29 - 0.31)
POLYUNSATPCaa	0.54	-0.17 (-0.46 - 0.12)	0.99	-0.01 (-0.31 - 0.29)
PCaa	0.53	-0.18 (-0.47 - 0.11)	0.99	-0.01 (-0.31 - 0.29)
SATURATEDPCae	0.41	-0.26 (-0.56 - 0.03)	0.94	-0.07 (-0.37 - 0.24)
MONOUNSATPCae	0.41	-0.27 (-0.56 - 0.03)	0.87	-0.16 (-0.46 - 0.14)
POLYUNSATPCae	0.48	-0.22 (-0.51 - 0.07)	0.87	-0.16 (-0.46 - 0.14)
PCae	0.45	-0.24 (-0.54 - 0.05)	0.89	-0.15 (-0.45 - 0.15)
SATURATEDLYSOPCa	0.54	0.17 (-0.13 - 0.46)	0.91	0.08 (-0.22 - 0.39)
MONOUNSATLYSOPCa	0.54	-0.17 (-0.48 - 0.14)	0.99	-0.03 (-0.34 - 0.28)
POLYUNSATLYSOPCa	0.62	-0.14 (-0.44 - 0.16)	0.89	0.11 (-0.21 - 0.42)
LYSOPCa	0.86	0.05 (-0.25 - 0.34)	0.92	0.08 (-0.23 - 0.38)
SATURATEDLYSOPCe	0.45	-0.23 (-0.52 - 0.07)	0.89	-0.13 (-0.44 - 0.17)
lyso.PC.e.C18.1	0.57	-0.15 (-0.44 - 0.15)	0.99	-0.01 (-0.3 - 0.29)
LYSOPCe	0.45	-0.22 (-0.51 - 0.07)	0.89	-0.12 (-0.42 - 0.18)
MONOUNSATSM	0.50	-0.2 (-0.5 - 0.09)	0.89	-0.12 (-0.42 - 0.18)
POLYUNSATSM	0.50	-0.2 (-0.49 - 0.09)	0.89	-0.13 (-0.43 - 0.18)
SM	0.50	-0.2 (-0.5 - 0.09)	0.89	-0.13 (-0.43 - 0.17)
Carn	0.44	0.25 (-0.04 - 0.54)	0.69	0.34 (0.05 - 0.63)
SHORTCHAINCARNa	0.71	0.1 (-0.19 - 0.39)	0.69	0.34 (0.05 - 0.64)
MEDIUMCHAINCARNa	0.83	-0.06 (-0.35 - 0.23)	0.89	0.11 (-0.2 - 0.41)
LONGCHAINCARNa	0.50	0.2 (-0.09 - 0.49)	0.89	0.14 (-0.16 - 0.44)
ACYLCARN	0.70	0.11 (-0.19 - 0.4)	0.69	0.32 (0.03 - 0.62)

Values represent absolute differences in SRS score and corresponding p-values from linear regression models that reflect the difference in SRS score at age 6 and 13 per SDS increase in cord-blood metabolite concentrations (µmol/L). Model includes sex and age at outcome. AA amino acids, NEFA non-esterified fatty acids, PC.aa diacyl-phosphatidylcholines, PC.ae acyl-alkyl-phosphatidylcholines, lyso.PC.a. acyl-lysophosphatidylcholines, lyso.PC.e alkyl-lysophosphatidylcholines, Carn.a acylcarnitines, SM sphingomyelines

Supplemental Table S5. Associations of cord-blood individual metabolites and metabolite groups with SRS scores at age 6 and 13. Adjusted model

	Differences in SRS score age 6 N = 716		Differences in SRS score age 13 N = 648	
Metabolite	P-value	Estimate (95%-Interval)	P-value	Estimate (95%-Interval)
Ala	1.00	0 (-0.3 - 0.3)	0.83	0.25 (-0.05 - 0.55)
Arg	0.45	-0.23 (-0.52 - 0.06)	0.98	0.03 (-0.27 - 0.34)
Asn	0.88	0.04 (-0.25 - 0.33)	0.71	0.28 (-0.02 - 0.58)
Asp	0.44	0.24 (-0.05 - 0.53)	0.71	0.29 (-0.02 - 0.59)
Cit	0.62	0.13 (-0.17 - 0.42)	0.84	0.19 (-0.11 - 0.49)
Gln	0.81	0.06 (-0.23 - 0.35)	0.93	0.14 (-0.16 - 0.45)
Glu	0.50	0.2 (-0.09 - 0.49)	0.84	0.21 (-0.09 - 0.5)
Gly	0.62	0.13 (-0.16 - 0.42)	0.84	0.19 (-0.11 - 0.5)
His	0.77	-0.07 (-0.37 - 0.22)	0.94	0.09 (-0.21 - 0.39)
Ile	0.77	0.08 (-0.22 - 0.37)	0.84	0.2 (-0.1 - 0.5)
Leu	0.86	0.04 (-0.25 - 0.33)	0.84	0.21 (-0.09 - 0.51)
Lys	0.88	0.04 (-0.26 - 0.33)	0.99	-0.03 (-0.34 - 0.28)
Met	0.98	0.01 (-0.28 - 0.3)	0.82	0.26 (-0.04 - 0.57)
Orn	0.33	0.31 (0.02 - 0.6)	0.83	0.25 (-0.05 - 0.55)
Phe	0.91	0.03 (-0.26 - 0.32)	0.93	0.13 (-0.18 - 0.44)
Pro	0.54	0.16 (-0.13 - 0.45)	0.84	0.22 (-0.08 - 0.52)
Trp	0.62	-0.14 (-0.43 - 0.15)	0.94	0.05 (-0.25 - 0.36)
Ser	0.45	0.23 (-0.06 - 0.52)	0.84	0.21 (-0.09 - 0.52)
Thr	0.86	-0.04 (-0.33 - 0.25)	0.84	0.23 (-0.07 - 0.53)
Tyr	0.97	0.01 (-0.28 - 0.31)	0.93	0.14 (-0.16 - 0.45)
Val	0.97	0.01 (-0.28 - 0.3)	0.84	0.2 (-0.1 - 0.5)
Cys	0.97	0.02 (-0.27 - 0.3)	0.99	-0.01 (-0.32 - 0.29)
NEFA_14_0	0.24	-0.36 (-0.64 - -0.07)	0.94	0.06 (-0.24 - 0.36)
NEFA_15_0	0.18	-0.39 (-0.68 - -0.1)	0.99	0 (-0.3 - 0.3)
NEFA_16_0	0.76	-0.08 (-0.37 - 0.21)	0.84	0.22 (-0.08 - 0.52)
NEFA_17_0	0.52	-0.19 (-0.48 - 0.1)	0.93	0.14 (-0.16 - 0.44)
NEFA_18_0	0.82	0.06 (-0.23 - 0.35)	0.71	0.29 (-0.01 - 0.59)
NEFA_24_0	0.75	-0.09 (-0.37 - 0.2)	0.82	0.26 (-0.03 - 0.56)
NEFA_26_0	0.45	-0.23 (-0.52 - 0.07)	0.84	0.19 (-0.11 - 0.48)
NEFA_14_1	0.18	-0.4 (-0.69 - -0.11)	0.99	-0.02 (-0.32 - 0.29)
NEFA_16_1	0.38	-0.29 (-0.58 - 0)	0.99	0.02 (-0.29 - 0.33)
NEFA_17_1	0.28	-0.33 (-0.62 - -0.04)	0.99	0.01 (-0.29 - 0.31)
NEFA_18_1	0.41	-0.26 (-0.55 - 0.03)	0.94	0.09 (-0.22 - 0.39)
NEFA_19_1	0.24	-0.36 (-0.65 - -0.07)	0.94	0.07 (-0.23 - 0.37)

NEFA_20_1	0.62	-0.14 (-0.43 - 0.15)	0.93	0.11 (-0.19 - 0.41)
NEFA_24_1	0.62	-0.13 (-0.42 - 0.16)	0.84	0.23 (-0.06 - 0.53)
NEFA_26_1	0.52	-0.19 (-0.48 - 0.1)	0.90	0.17 (-0.14 - 0.47)
NEFA_16_2	0.42	-0.26 (-0.55 - 0.03)	0.93	0.11 (-0.19 - 0.41)
NEFA_17_2	0.36	-0.3 (-0.59 - 0)	0.94	-0.05 (-0.35 - 0.25)
NEFA_18_2	0.53	-0.18 (-0.47 - 0.11)	0.93	0.15 (-0.15 - 0.45)
NEFA_18_3	0.38	-0.28 (-0.57 - 0.01)	0.94	0.09 (-0.21 - 0.38)
NEFA_20_2	0.84	-0.05 (-0.35 - 0.24)	0.86	0.18 (-0.12 - 0.48)
NEFA_20_3	0.64	-0.12 (-0.41 - 0.17)	0.94	0.06 (-0.23 - 0.35)
NEFA_20_4	0.43	-0.25 (-0.54 - 0.04)	0.99	0 (-0.29 - 0.3)
NEFA_20_5	0.28	-0.34 (-0.63 - -0.05)	0.84	-0.21 (-0.52 - 0.09)
NEFA_22_3	0.97	0.01 (-0.28 - 0.3)	0.89	0.17 (-0.12 - 0.46)
NEFA_22_4	0.58	-0.15 (-0.45 - 0.14)	0.94	0.09 (-0.21 - 0.4)
NEFA_22_5	0.62	-0.13 (-0.42 - 0.16)	0.99	0.02 (-0.28 - 0.32)
NEFA_22_6	0.53	-0.17 (-0.47 - 0.12)	0.94	0.05 (-0.25 - 0.35)
NEFA_24_2	0.81	-0.06 (-0.35 - 0.23)	0.71	0.34 (0.04 - 0.64)
NEFA_24_4	0.62	0.13 (-0.16 - 0.42)	0.71	0.29 (-0.01 - 0.59)
NEFA_24_5	0.96	-0.02 (-0.31 - 0.27)	0.84	0.2 (-0.09 - 0.5)
NEFA_26_2	0.85	-0.05 (-0.34 - 0.24)	0.84	0.23 (-0.07 - 0.53)
PC.aa.C30.0	0.18	-0.41 (-0.71 - -0.11)	0.94	-0.05 (-0.36 - 0.25)
PC.aa.C32.0	0.40	-0.28 (-0.58 - 0.02)	0.93	-0.15 (-0.46 - 0.16)
PC.aa.C36.0	0.83	-0.05 (-0.34 - 0.23)	0.99	0.02 (-0.29 - 0.32)
PC.aa.C38.0	0.67	-0.12 (-0.42 - 0.18)	0.94	-0.08 (-0.39 - 0.22)
PC.aa.C40.0	0.75	-0.09 (-0.38 - 0.21)	0.93	-0.14 (-0.44 - 0.16)
PC.aa.C42.0	0.62	-0.14 (-0.44 - 0.17)	0.93	-0.12 (-0.43 - 0.2)
PC.aa.C32.1	0.33	-0.33 (-0.63 - -0.03)	0.99	-0.03 (-0.33 - 0.28)
PC.aa.C34.1	0.57	-0.16 (-0.46 - 0.14)	0.99	0.02 (-0.28 - 0.32)
PC.aa.C36.1	0.48	-0.22 (-0.51 - 0.08)	0.96	0.04 (-0.26 - 0.34)
PC.aa.C40.1	0.67	-0.12 (-0.41 - 0.17)	0.83	-0.25 (-0.55 - 0.05)
PC.aa.C30.3	0.38	-0.28 (-0.57 - 0.01)	0.94	-0.09 (-0.39 - 0.21)
PC.aa.C32.2	0.86	-0.05 (-0.34 - 0.24)	0.71	-0.38 (-0.68 - -0.08)
PC.aa.C32.3	0.75	-0.09 (-0.38 - 0.2)	0.84	0.19 (-0.11 - 0.49)
PC.aa.C34.2	0.70	-0.11 (-0.4 - 0.19)	0.94	0.1 (-0.2 - 0.4)
PC.aa.C34.3	0.43	-0.26 (-0.55 - 0.04)	0.98	0.04 (-0.26 - 0.34)
PC.aa.C34.4	0.45	-0.23 (-0.52 - 0.06)	0.94	0.07 (-0.23 - 0.37)
PC.aa.C34.5	0.14	-0.45 (-0.75 - -0.16)	0.94	-0.09 (-0.39 - 0.21)
PC.aa.C36.2	0.77	-0.08 (-0.37 - 0.22)	0.93	0.15 (-0.15 - 0.46)
PC.aa.C36.3	0.55	-0.16 (-0.46 - 0.13)	0.99	-0.02 (-0.32 - 0.29)
PC.aa.C36.4	0.50	-0.2 (-0.49 - 0.09)	0.94	-0.05 (-0.35 - 0.26)
PC.aa.C36.5	0.24	-0.37 (-0.66 - -0.07)	0.94	-0.08 (-0.39 - 0.23)
PC.aa.C36.6	0.57	-0.16 (-0.44 - 0.13)	0.99	0 (-0.3 - 0.3)

PC.aa.C38.2	0.49	-0.21 (-0.5 - 0.08)	0.93	-0.13 (-0.42 - 0.17)
PC.aa.C38.3	0.68	-0.11 (-0.41 - 0.18)	0.98	0.04 (-0.27 - 0.34)
PC.aa.C38.4	0.58	-0.15 (-0.44 - 0.14)	0.99	-0.02 (-0.32 - 0.28)
PC.aa.C38.5	0.67	-0.12 (-0.41 - 0.18)	0.99	0 (-0.3 - 0.3)
PC.aa.C38.6	0.62	-0.14 (-0.44 - 0.16)	0.94	-0.09 (-0.41 - 0.22)
PC.aa.C40.2	0.53	-0.18 (-0.47 - 0.11)	0.71	-0.32 (-0.62 - -0.02)
PC.aa.C40.3	0.45	-0.23 (-0.52 - 0.06)	0.71	-0.32 (-0.61 - -0.02)
PC.aa.C40.4	0.98	0.01 (-0.29 - 0.3)	0.99	0.01 (-0.29 - 0.32)
PC.aa.C40.5	0.74	-0.1 (-0.4 - 0.2)	0.94	-0.05 (-0.36 - 0.25)
PC.aa.C40.6	0.86	-0.05 (-0.36 - 0.26)	0.99	0 (-0.33 - 0.32)
PC.aa.C42.5	0.62	-0.13 (-0.43 - 0.17)	0.93	-0.14 (-0.44 - 0.17)
PC.aa.C43.6	0.78	0.07 (-0.23 - 0.37)	0.93	0.13 (-0.18 - 0.44)
PC.aa.C44.12	0.50	-0.21 (-0.51 - 0.09)	0.94	-0.08 (-0.39 - 0.24)
PC.ae.C30.0	0.49	-0.21 (-0.5 - 0.08)	0.71	-0.28 (-0.58 - 0.01)
PC.ae.C32.0	0.52	-0.19 (-0.49 - 0.1)	0.86	-0.18 (-0.48 - 0.12)
PC.ae.C34.0	0.18	-0.4 (-0.71 - -0.1)	0.84	-0.2 (-0.5 - 0.11)
PC.ae.C36.0	0.99	0 (-0.3 - 0.29)	0.99	0 (-0.3 - 0.31)
PC.ae.C38.0	0.45	-0.23 (-0.52 - 0.06)	0.93	-0.15 (-0.46 - 0.15)
PC.ae.C40.0	0.45	-0.22 (-0.52 - 0.07)	0.94	0.05 (-0.25 - 0.36)
PC.ae.C32.1	0.52	-0.19 (-0.49 - 0.11)	0.98	-0.04 (-0.35 - 0.27)
PC.ae.C34.1	0.33	-0.32 (-0.62 - -0.02)	0.93	-0.11 (-0.42 - 0.19)
PC.ae.C36.1	0.50	-0.2 (-0.5 - 0.1)	0.84	-0.24 (-0.54 - 0.07)
PC.ae.C40.1	0.75	-0.09 (-0.39 - 0.21)	0.94	-0.09 (-0.39 - 0.21)
PC.ae.C42.1	0.99	0 (-0.29 - 0.29)	0.94	-0.11 (-0.41 - 0.2)
PC.ae.C32.2	0.14	-0.44 (-0.74 - -0.15)	0.71	-0.3 (-0.61 - 0)
PC.ae.C34.2	0.45	-0.23 (-0.52 - 0.07)	0.94	-0.09 (-0.39 - 0.22)
PC.ae.C34.3	0.45	-0.24 (-0.54 - 0.05)	0.99	-0.03 (-0.33 - 0.27)
PC.ae.C34.4	0.15	-0.42 (-0.71 - -0.14)	0.71	-0.43 (-0.73 - -0.13)
PC.ae.C36.2	0.45	-0.24 (-0.53 - 0.06)	0.90	-0.16 (-0.46 - 0.13)
PC.ae.C36.3	0.77	-0.08 (-0.37 - 0.22)	0.95	-0.04 (-0.34 - 0.25)
PC.ae.C36.4	0.50	-0.2 (-0.5 - 0.09)	0.85	-0.18 (-0.48 - 0.12)
PC.ae.C36.5	0.62	-0.13 (-0.42 - 0.17)	0.89	-0.17 (-0.48 - 0.13)
PC.ae.C38.2	0.28	-0.33 (-0.62 - -0.04)	0.84	-0.21 (-0.51 - 0.08)
PC.ae.C38.3	0.40	-0.27 (-0.56 - 0.02)	0.93	-0.15 (-0.45 - 0.15)
PC.ae.C38.4	0.57	-0.16 (-0.45 - 0.14)	0.94	-0.07 (-0.37 - 0.24)
PC.ae.C38.5	0.62	-0.14 (-0.43 - 0.16)	0.94	-0.08 (-0.38 - 0.22)
PC.ae.C38.6	0.58	-0.15 (-0.44 - 0.14)	0.84	-0.2 (-0.5 - 0.1)
PC.ae.C40.2	0.82	-0.06 (-0.35 - 0.23)	0.99	-0.02 (-0.32 - 0.28)
PC.ae.C40.3	0.38	-0.28 (-0.58 - 0.01)	0.98	-0.04 (-0.34 - 0.27)
PC.ae.C40.4	0.28	-0.35 (-0.65 - -0.04)	0.93	-0.13 (-0.44 - 0.18)
PC.ae.C40.5	0.38	-0.29 (-0.58 - 0.01)	0.83	-0.25 (-0.55 - 0.05)

PC.ae.C40.6	0.45	-0.25 (-0.56 - 0.05)	0.94	-0.08 (-0.4 - 0.24)
PC.ae.C42.3	0.71	-0.1 (-0.39 - 0.19)	0.94	-0.1 (-0.4 - 0.19)
PC.ae.C42.4	0.57	-0.15 (-0.44 - 0.14)	0.94	-0.08 (-0.38 - 0.21)
PC.ae.C42.5	0.75	-0.09 (-0.39 - 0.21)	0.99	-0.02 (-0.33 - 0.28)
PC.ae.C42.6	0.45	-0.25 (-0.55 - 0.06)	0.93	-0.16 (-0.48 - 0.15)
lyso.PC.a.C14.0	0.71	-0.1 (-0.39 - 0.19)	0.96	0.04 (-0.26 - 0.34)
lyso.PC.a.C16.0	0.53	0.18 (-0.12 - 0.47)	0.94	0.09 (-0.22 - 0.39)
lyso.PC.a.C18.0	0.53	0.18 (-0.11 - 0.47)	0.94	0.07 (-0.24 - 0.38)
lyso.PC.a.C16.1	0.62	-0.14 (-0.44 - 0.16)	0.94	0.06 (-0.24 - 0.37)
lyso.PC.a.C18.1	0.53	-0.18 (-0.49 - 0.12)	0.94	-0.07 (-0.38 - 0.24)
lyso.PC.a.C18.2	0.76	-0.08 (-0.39 - 0.22)	0.84	0.21 (-0.11 - 0.52)
lyso.PC.a.C18.3	0.89	0.03 (-0.26 - 0.33)	0.84	0.2 (-0.1 - 0.5)
lyso.PC.a.C20.3	0.70	-0.11 (-0.41 - 0.19)	0.94	0.05 (-0.26 - 0.36)
lyso.PC.a.C20.4	0.57	-0.16 (-0.45 - 0.14)	0.94	0.07 (-0.24 - 0.38)
lyso.PC.a.C20.5	0.40	-0.27 (-0.57 - 0.02)	0.94	-0.06 (-0.36 - 0.25)
lyso.PC.a.C22.6	0.55	-0.17 (-0.46 - 0.13)	0.90	-0.17 (-0.48 - 0.14)
lyso.PC.e.C16.0	0.60	-0.14 (-0.43 - 0.15)	0.94	-0.1 (-0.39 - 0.19)
lyso.PC.e.C18.0	0.45	-0.24 (-0.53 - 0.06)	0.93	-0.12 (-0.43 - 0.18)
lyso.PC.e.C18.1	0.57	-0.16 (-0.45 - 0.14)	0.99	-0.01 (-0.31 - 0.29)
SM.a.C30.1	0.43	-0.25 (-0.54 - 0.04)	0.93	-0.13 (-0.43 - 0.17)
SM.a.C32.1	0.28	-0.33 (-0.63 - -0.04)	0.93	-0.13 (-0.43 - 0.17)
SM.a.C33.1	0.30	-0.32 (-0.61 - -0.03)	0.84	-0.24 (-0.54 - 0.06)
SM.a.C34.1	0.53	-0.18 (-0.48 - 0.11)	0.93	-0.12 (-0.42 - 0.18)
SM.a.C35.1	0.82	-0.06 (-0.35 - 0.23)	0.99	-0.01 (-0.31 - 0.29)
SM.a.C36.1	0.53	-0.18 (-0.47 - 0.11)	0.94	-0.05 (-0.35 - 0.25)
SM.a.C37.1	0.44	-0.25 (-0.54 - 0.04)	0.94	-0.05 (-0.35 - 0.24)
SM.a.C39.1	0.36	-0.3 (-0.59 - 0)	0.94	-0.05 (-0.35 - 0.25)
SM.a.C41.1	0.18	-0.4 (-0.69 - -0.11)	0.93	-0.14 (-0.44 - 0.17)
SM.a.C42.1	0.76	-0.08 (-0.38 - 0.21)	0.94	-0.09 (-0.39 - 0.21)
SM.a.C43.1	0.28	-0.34 (-0.64 - -0.05)	0.93	-0.15 (-0.45 - 0.15)
SM.a.C32.2	0.36	-0.29 (-0.58 - 0)	0.94	-0.08 (-0.39 - 0.22)
SM.a.C34.2	0.54	-0.17 (-0.46 - 0.12)	0.94	-0.07 (-0.37 - 0.23)
SM.a.C36.2	0.52	-0.19 (-0.48 - 0.11)	0.99	0.03 (-0.28 - 0.33)
SM.a.C36.3	0.75	-0.09 (-0.38 - 0.2)	0.99	-0.02 (-0.32 - 0.28)
SM.a.C38.2	0.62	-0.14 (-0.43 - 0.16)	0.94	-0.07 (-0.39 - 0.24)
SM.a.C38.3	0.77	-0.08 (-0.37 - 0.22)	0.99	0.01 (-0.29 - 0.32)
SM.a.C39.2	0.08	-0.54 (-0.84 - -0.24)	0.94	0.05 (-0.25 - 0.35)
SM.a.C40.2	0.53	-0.18 (-0.47 - 0.11)	0.93	-0.13 (-0.43 - 0.18)
SM.a.C40.5	0.50	-0.2 (-0.49 - 0.1)	0.90	-0.17 (-0.48 - 0.14)
SM.a.C41.2	0.50	-0.2 (-0.49 - 0.1)	0.93	-0.11 (-0.41 - 0.18)
SM.a.C42.2	0.48	-0.22 (-0.52 - 0.07)	0.84	-0.19 (-0.48 - 0.11)

SM.a.C42.3	0.76	-0.08 (-0.37 - 0.21)	0.94	-0.1 (-0.4 - 0.21)
SM.a.C42.4	0.53	-0.17 (-0.47 - 0.12)	0.99	-0.03 (-0.33 - 0.27)
SM.a.C42.6	0.54	-0.17 (-0.47 - 0.13)	0.94	-0.1 (-0.41 - 0.21)
SM.a.C43.2	0.33	-0.31 (-0.6 - -0.02)	0.99	0 (-0.31 - 0.3)
SM.a.C44.6	0.52	-0.2 (-0.49 - 0.1)	0.94	-0.06 (-0.37 - 0.26)
SM.e.C36.2	0.62	-0.13 (-0.43 - 0.16)	0.94	-0.07 (-0.37 - 0.23)
SM.e.C38.3	0.49	-0.21 (-0.5 - 0.08)	0.99	-0.01 (-0.31 - 0.29)
SM.e.C40.5	0.76	-0.08 (-0.37 - 0.21)	0.94	-0.11 (-0.41 - 0.19)
Carn.a.C2.0	0.64	0.12 (-0.17 - 0.42)	0.71	0.37 (0.08 - 0.66)
Carn.a.C3.0	0.97	0.02 (-0.28 - 0.31)	0.93	0.14 (-0.16 - 0.43)
Carn.a.C3.0.DC	0.70	-0.11 (-0.4 - 0.19)	0.94	0.07 (-0.23 - 0.37)
Carn.a.C4.0	0.75	-0.09 (-0.39 - 0.21)	0.94	0.08 (-0.22 - 0.39)
Carn.a.C5.0	0.97	0.01 (-0.28 - 0.31)	0.94	-0.05 (-0.36 - 0.26)
Carn.a.C6.0	0.97	-0.01 (-0.31 - 0.29)	0.84	0.25 (-0.06 - 0.56)
Carn.a.C6.0.OH	0.79	0.07 (-0.22 - 0.35)	0.94	0.05 (-0.25 - 0.35)
Carn.a.C8.0	0.73	-0.1 (-0.39 - 0.2)	0.94	0.08 (-0.22 - 0.37)
Carn.a.C8.1	0.62	-0.13 (-0.43 - 0.17)	0.90	-0.17 (-0.48 - 0.14)
Carn.a.C9.0	0.77	0.07 (-0.22 - 0.37)	0.93	0.14 (-0.16 - 0.45)
Carn.a.C10.0	0.53	-0.17 (-0.47 - 0.12)	0.94	0.07 (-0.24 - 0.37)
Carn.a.C10.1	0.77	0.07 (-0.22 - 0.37)	0.84	0.2 (-0.11 - 0.5)
Carn.a.C12.0	0.91	-0.03 (-0.32 - 0.26)	0.93	0.13 (-0.18 - 0.43)
Carn.a.C14.1	0.86	-0.04 (-0.33 - 0.25)	0.94	0.07 (-0.24 - 0.37)
Carn.a.C14.2	0.71	0.1 (-0.19 - 0.39)	0.84	0.21 (-0.1 - 0.52)
Carn.a.C15.0	0.85	0.05 (-0.24 - 0.35)	0.84	0.21 (-0.09 - 0.52)
Carn.a.C16.0	0.36	0.3 (0.01 - 0.6)	0.93	0.12 (-0.19 - 0.42)
Carn.a.C16.0.Oxo	0.88	0.04 (-0.25 - 0.33)	0.99	0.02 (-0.28 - 0.32)
Carn.a.C16.1	0.58	0.15 (-0.14 - 0.44)	0.84	0.21 (-0.1 - 0.51)
Carn.a.C16.2	0.78	0.07 (-0.22 - 0.36)	0.98	0.04 (-0.27 - 0.34)
Carn.a.C18.0	0.86	0.04 (-0.25 - 0.33)	0.94	0.1 (-0.2 - 0.39)
Carn.a.C18.1	0.53	0.17 (-0.12 - 0.47)	0.99	0.02 (-0.28 - 0.32)
Carn.a.C18.2	0.08	0.51 (0.22 - 0.8)	0.71	0.32 (0.01 - 0.62)
Carn.a.C18.2.OH	0.77	0.08 (-0.22 - 0.37)	0.99	0.02 (-0.28 - 0.33)
Carn.a.C20.0	0.86	0.04 (-0.24 - 0.33)	0.94	0.09 (-0.2 - 0.39)
Carn.a.C20.1	0.74	0.09 (-0.2 - 0.39)	0.84	0.19 (-0.1 - 0.49)
Carn.a.C20.3	0.68	0.11 (-0.18 - 0.4)	0.94	0.06 (-0.24 - 0.36)
Carn.a.C20.4	0.75	0.08 (-0.2 - 0.37)	0.84	0.19 (-0.11 - 0.5)
BCAA	0.89	0.03 (-0.26 - 0.33)	0.84	0.22 (-0.09 - 0.52)
AAA	0.88	-0.04 (-0.33 - 0.26)	0.93	0.12 (-0.18 - 0.42)
EAA	1.00	0 (-0.29 - 0.29)	0.86	0.18 (-0.12 - 0.49)
NEAA	0.62	0.14 (-0.15 - 0.43)	0.71	0.29 (-0.01 - 0.6)
AA	0.76	0.08 (-0.21 - 0.37)	0.82	0.26 (-0.04 - 0.56)

SATURATEDNEFA	0.74	-0.09 (-0.39 - 0.2)	0.84	0.23 (-0.08 - 0.53)
MONOUNSATNEFA	0.38	-0.28 (-0.57 - 0.01)	0.94	0.07 (-0.23 - 0.37)
POLYUNSATNEFA	0.49	-0.21 (-0.5 - 0.08)	0.93	0.12 (-0.18 - 0.42)
NEFA	0.52	-0.18 (-0.48 - 0.11)	0.93	0.15 (-0.15 - 0.45)
SATURATEDPCaa	0.38	-0.29 (-0.59 - 0.01)	0.93	-0.13 (-0.44 - 0.18)
MONOUNSATPCaa	0.52	-0.19 (-0.49 - 0.11)	0.99	0.02 (-0.28 - 0.32)
POLYUNSATPCaa	0.54	-0.17 (-0.46 - 0.12)	0.99	0 (-0.31 - 0.3)
PCaa	0.53	-0.18 (-0.47 - 0.11)	0.99	0 (-0.31 - 0.3)
SATURATEDPCae	0.41	-0.26 (-0.56 - 0.03)	0.94	-0.05 (-0.35 - 0.26)
MONOUNSATPCae	0.41	-0.27 (-0.57 - 0.03)	0.93	-0.14 (-0.44 - 0.16)
POLYUNSATPCae	0.48	-0.22 (-0.51 - 0.08)	0.93	-0.15 (-0.45 - 0.15)
PCae	0.45	-0.24 (-0.54 - 0.05)	0.93	-0.14 (-0.44 - 0.16)
SATURATEDLYSOPCa	0.54	0.17 (-0.13 - 0.46)	0.94	0.08 (-0.22 - 0.39)
MONOUNSATLYSOPCa	0.54	-0.17 (-0.48 - 0.13)	0.99	-0.03 (-0.34 - 0.28)
POLYUNSATLYSOPCa	0.62	-0.14 (-0.44 - 0.16)	0.94	0.11 (-0.2 - 0.42)
LYSOPCa	0.86	0.05 (-0.25 - 0.34)	0.94	0.08 (-0.23 - 0.39)
SATURATEDLYSOPCe	0.45	-0.22 (-0.52 - 0.07)	0.93	-0.13 (-0.43 - 0.17)
lyso.PC.e.C18.1	0.57	-0.16 (-0.45 - 0.14)	0.99	-0.01 (-0.31 - 0.29)
LYSOPCe	0.45	-0.22 (-0.51 - 0.07)	0.93	-0.12 (-0.42 - 0.18)
MONOUNSATSM	0.50	-0.2 (-0.5 - 0.09)	0.93	-0.11 (-0.41 - 0.19)
POLYUNSATSM	0.50	-0.2 (-0.49 - 0.09)	0.93	-0.12 (-0.42 - 0.19)
SM	0.50	-0.2 (-0.5 - 0.09)	0.93	-0.12 (-0.42 - 0.19)
Carn	0.44	0.25 (-0.05 - 0.54)	0.71	0.34 (0.05 - 0.63)
SHORTCHAINCARNa	0.71	0.1 (-0.19 - 0.4)	0.71	0.34 (0.05 - 0.64)
MEDIUMCHAINCARNa	0.83	-0.06 (-0.35 - 0.24)	0.93	0.12 (-0.19 - 0.42)
LONGCHAINCARNa	0.50	0.2 (-0.1 - 0.49)	0.93	0.15 (-0.15 - 0.46)
ACYLCARN	0.70	0.11 (-0.18 - 0.4)	0.71	0.32 (0.03 - 0.62)

Values represent absolute differences in SRS score and corresponding p-values from linear regression models that reflect the difference in SRS score at age 6 and 13 per SDS increase in cord-blood metabolite concentrations (µmol/L). Model includes sex and age at outcome. AA amino acids, NEFA non-esterified fatty acids, PC.aa diacyl-phosphatidylcholines, PC.ae acyl-alkyl-phosphatidylcholines, lyso.PC.a. acyl-lysophosphatidylcholines, lyso.PC.e alkyl-lysophosphatidylcholines, Carn.a acylcarnitines, SM sphingomyelines

Supplemental Table S6. Associations of cord blood metabolite ratios with SRS scores at age 6 and 13. Basic model.

	Difference in SRS score age 6		Difference in SRS score age 13	
Metabolite ratios	P-value	Estimate (95%-Interval)	P-value	Estimate (95%-Interval)
Asn/Asp	0.33	-0.22 (-0.51 - 0.07)	0.76	-0.11 (-0.4 - 0.18)
Gln/Glu	0.54	-0.14 (-0.44 - 0.15)	0.66	-0.16 (-0.46 - 0.14)
NEFA.18:1/18:0	0.20	-0.3 (-0.59 - -0.02)	0.51	-0.25 (-0.55 - 0.05)
NEFA.16:1/16:0	0.02	-0.55 (-0.83 - -0.26)	0.40	-0.38 (-0.69 - -0.08)
Carn.a.16.0/free carnitine	0.26	0.28 (-0.02 - 0.58)	0.33	0.46 (0.16 - 0.76)
Carn.a.C:2/C:16	0.11	0.38 (0.08 - 0.67)	0.63	0.18 (-0.12 - 0.47)
∑Lyso.PC.a/∑PC.aa	0.06	0.45 (0.15 - 0.74)	0.70	0.15 (-0.15 - 0.44)
(Lyso.PC.a.C:18.1 + C:18.2)/∑PC.aa	0.43	0.18 (-0.11 - 0.48)	0.54	0.22 (-0.07 - 0.52)
(Lyso.PC.a.C:16.0 + C:18.0)/∑PC.aa	0.81	-0.06 (-0.35 - 0.23)	0.40	-0.32 (-0.61 - -0.03)
Val/PC.ae.C:32.2	0.81	-0.06 (-0.36 - 0.24)	0.40	0.34 (0.04 - 0.63)
∑PC.aa/∑PC.ae	0.11	0.37 (0.08 - 0.66)	0.40	0.36 (0.07 - 0.66)

Supplemental(1) Table S7. Associations of cord blood metabolite ratios with SRS scores at age 6 and 13. Main model.

	Difference in SRS score age 6		Difference in SRS score age 13	
Metabolite ratios	P-value	Estimate (95%-Interval)	P-value	Estimate (95%-Interval)
Asn/Asp	0.43	-0.24 (-0.53 - 0.04)	0.89	-0.11 (-0.4 - 0.18)
Gln/Glu	0.62	-0.13 (-0.42 - 0.16)	0.89	-0.12 (-0.42 - 0.17)
NEFA.18:1/18:0	0.37	-0.28 (-0.56 - 0.01)	0.75	-0.26 (-0.56 - 0.04)
NEFA.16:1/16:0	0.08	-0.48 (-0.77 - -0.19)	0.69	-0.32 (-0.63 - -0.01)
Carn.a.16.0/free carnitine	0.51	0.2 (-0.1 - 0.49)	0.69	0.36 (0.06 - 0.67)
Carn.a.C:2/C:16	0.37	0.28 (-0.02 - 0.58)	0.89	0.1 (-0.19 - 0.4)
∑Lyso.PC.a/∑PC.aa	0.18	0.41 (0.12 - 0.7)	0.89	0.11 (-0.19 - 0.4)
(Lyso.PC.a.C:18.1 + C:18.2)/∑PC.aa	0.97	0.01 (-0.3 - 0.32)	0.90	0.09 (-0.22 - 0.4)
(Lyso.PC.a.C:16.0 + C:18.0)/∑PC.aa	0.97	-0.01 (-0.3 - 0.28)	0.75	-0.26 (-0.55 - 0.03)
Val/PC.ae.C:32.2	0.73	-0.1 (-0.39 - 0.2)	0.69	0.31 (0.02 - 0.61)
∑PC.aa/∑PC.ae	0.24	0.36 (0.07 - 0.65)	0.69	0.33 (0.03 - 0.63)

Supplemental Table S8. Associations of cord blood metabolite ratios with SRS scores at age 6 and 13. Adjusted model.

	Difference in SRS score age 6		Difference in SRS score age 13	
**Metabolite ratios**	**P-value**	**Estimate (95%-Interval)**	**P-value**	**Estimate (95%-Interval)**
Asn/Asp	0.44	-0.24 (-0.53 - 0.04)	0.94	-0.1 (-0.4 - 0.19)
Gln/Glu	0.62	-0.13 (-0.42 - 0.16)	0.93	-0.11 (-0.41 - 0.19)
NEFA.18:1/18:0	0.38	-0.28 (-0.57 - 0)	0.82	-0.26 (-0.56 - 0.04)
NEFA.16:1/16:0	0.08	-0.48 (-0.77 - -0.19)	0.71	-0.32 (-0.63 - -0.01)
Carn.a.16.0/free carnitine	0.52	0.19 (-0.1 - 0.49)	0.71	0.36 (0.06 - 0.66)
Carn.a.C:2/C:16	0.38	0.28 (-0.02 - 0.58)	0.94	0.09 (-0.2 - 0.39)
∑Lyso.PC.a/∑PC.aa	0.18	0.41 (0.12 - 0.7)	0.94	0.1 (-0.2 - 0.39)
(Lyso.PC.a.C:18.1 + C:18.2)/∑PC.aa	0.97	0.01 (-0.3 - 0.32)	0.94	0.08 (-0.23 - 0.39)
(Lyso.PC.a.C:16.0 + C:18.0)/∑PC.aa	0.98	-0.01 (-0.3 - 0.28)	0.82	-0.25 (-0.54 - 0.03)
Val/PC.ae.C:32.2	0.75	-0.09 (-0.39 - 0.2)	0.71	0.31 (0.01 - 0.6)
∑PC.aa/∑PC.ae	0.24	0.36 (0.07 - 0.65)	0.71	0.32 (0.02 - 0.62)

Supplemental Table S9. P-values for interaction effects of linear mixed-effects models.

Metabolite	inter_p	fdr_inter_p
Ala	0.08	0.83
Arg	0.09	0.88
Asn	0.05	0.69
Asp	0.31	1.00
Cit	0.37	1.00
Gln	0.57	1.00
Glu	0.94	1.00
Gly	0.50	1.00
His	0.21	1.00
Ile	0.80	1.00
Leu	0.33	1.00
Lys	0.80	1.00
Met	0.03	0.69
Orn	0.94	1.00
Phe	0.35	1.00
Pro	0.44	1.00
Trp	0.36	1.00
Ser	0.70	1.00
Thr	0.23	1.00
Tyr	0.41	1.00
Val	0.22	1.00
Cys	0.93	1.00
NEFA_14_0	0.01	0.69
NEFA_15_0	0.05	0.69
NEFA_16_0	0.04	0.69
NEFA_17_0	0.06	0.71
NEFA_18_0	0.16	1.00
NEFA_24_0	0.05	0.69
NEFA_26_0	0.10	0.89
NEFA_14_1	0.03	0.69
NEFA_16_1	0.10	0.89
NEFA_17_1	0.09	0.84
NEFA_18_1	0.04	0.69
NEFA_19_1	0.02	0.69
NEFA_20_1	0.32	1.00
NEFA_24_1	0.08	0.83
NEFA_26_1	0.17	1.00
NEFA_16_2	0.05	0.69
NEFA_17_2	0.22	1.00

NEFA_18_2	0.05	0.69
NEFA_18_3	0.07	0.78
NEFA_20_2	0.11	0.91
NEFA_20_3	0.30	1.00
NEFA_20_4	0.20	1.00
NEFA_20_5	0.75	1.00
NEFA_22_3	0.29	1.00
NEFA_22_4	0.15	1.00
NEFA_22_5	0.28	1.00
NEFA_22_6	0.19	1.00
NEFA_24_2	0.02	0.69
NEFA_24_4	0.24	1.00
NEFA_24_5	0.18	1.00
NEFA_26_2	0.23	1.00
PC.aa.C30.0	0.26	1.00
PC.aa.C32.0	0.74	1.00
PC.aa.C36.0	0.83	1.00
PC.aa.C38.0	0.75	1.00
PC.aa.C40.0	0.81	1.00
PC.aa.C42.0	0.30	1.00
PC.aa.C32.1	0.77	1.00
PC.aa.C34.1	0.95	1.00
PC.aa.C36.1	0.62	1.00
PC.aa.C40.1	0.42	1.00
PC.aa.C30.3	0.53	1.00
PC.aa.C32.2	0.00	0.69
PC.aa.C32.3	0.66	1.00
PC.aa.C34.2	0.69	1.00
PC.aa.C34.3	0.73	1.00
PC.aa.C34.4	0.99	1.00
PC.aa.C34.5	0.12	1.00
PC.aa.C36.2	0.61	1.00
PC.aa.C36.3	0.92	1.00
PC.aa.C36.4	0.98	1.00
PC.aa.C36.5	0.43	1.00
PC.aa.C36.6	0.50	1.00
PC.aa.C38.2	0.43	1.00
PC.aa.C38.3	0.71	1.00
PC.aa.C38.4	0.87	1.00
PC.aa.C38.5	0.72	1.00
PC.aa.C38.6	0.82	1.00
PC.aa.C40.2	0.39	1.00

PC.aa.C40.3	0.70	1.00
PC.aa.C40.4	0.94	1.00
PC.aa.C40.5	0.74	1.00
PC.aa.C40.6	0.99	1.00
PC.aa.C42.5	0.80	1.00
PC.aa.C43.6	0.45	1.00
PC.aa.C44.12	0.46	1.00
PC.ae.C30.0	0.55	1.00
PC.ae.C32.0	0.59	1.00
PC.ae.C34.0	0.83	1.00
PC.ae.C36.0	0.70	1.00
PC.ae.C38.0	0.86	1.00
PC.ae.C40.0	0.32	1.00
PC.ae.C32.1	0.70	1.00
PC.ae.C34.1	0.89	1.00
PC.ae.C36.1	0.21	1.00
PC.ae.C40.1	0.40	1.00
PC.ae.C42.1	1.00	1.00
PC.ae.C32.2	0.79	1.00
PC.ae.C34.2	0.89	1.00
PC.ae.C34.3	0.46	1.00
PC.ae.C34.4	0.75	1.00
PC.ae.C36.2	0.79	1.00
PC.ae.C36.3	0.78	1.00
PC.ae.C36.4	0.88	1.00
PC.ae.C36.5	0.47	1.00
PC.ae.C38.2	0.53	1.00
PC.ae.C38.3	0.75	1.00
PC.ae.C38.4	0.77	1.00
PC.ae.C38.5	0.88	1.00
PC.ae.C38.6	1.00	1.00
PC.ae.C40.2	0.61	1.00
PC.ae.C40.3	0.22	1.00
PC.ae.C40.4	0.46	1.00
PC.ae.C40.5	0.72	1.00
PC.ae.C40.6	0.36	1.00
PC.ae.C42.3	0.56	1.00
PC.ae.C42.4	0.33	1.00
PC.ae.C42.5	0.90	1.00
PC.ae.C42.6	0.78	1.00
lyso.PC.a.C14.0	0.55	1.00
lyso.PC.a.C16.0	0.28	1.00

lyso.PC.a.C18.0	0.60	1.00
lyso.PC.a.C16.1	0.49	1.00
lyso.PC.a.C18.1	0.70	1.00
lyso.PC.a.C18.2	0.60	1.00
lyso.PC.a.C18.3	0.64	1.00
lyso.PC.a.C20.3	0.81	1.00
lyso.PC.a.C20.4	0.62	1.00
lyso.PC.a.C20.5	0.30	1.00
lyso.PC.a.C22.6	0.72	1.00
lyso.PC.e.C16.0	0.30	1.00
lyso.PC.e.C18.0	0.49	1.00
lyso.PC.e.C18.1	1.00	1.00
SM.a.C30.1	0.61	1.00
SM.a.C32.1	0.93	1.00
SM.a.C33.1	0.75	1.00
SM.a.C34.1	0.70	1.00
SM.a.C35.1	0.71	1.00
SM.a.C36.1	0.82	1.00
SM.a.C37.1	0.18	1.00
SM.a.C39.1	0.30	1.00
SM.a.C41.1	0.58	1.00
SM.a.C42.1	0.61	1.00
SM.a.C43.1	0.31	1.00
SM.a.C32.2	0.79	1.00
SM.a.C34.2	0.87	1.00
SM.a.C36.2	0.79	1.00
SM.a.C36.3	0.35	1.00
SM.a.C38.2	0.75	1.00
SM.a.C38.3	0.51	1.00
SM.a.C39.2	0.02	0.69
SM.a.C40.2	0.91	1.00
SM.a.C40.5	0.29	1.00
SM.a.C41.2	0.61	1.00
SM.a.C42.2	0.63	1.00
SM.a.C42.3	0.52	1.00
SM.a.C42.4	0.91	1.00
SM.a.C42.6	0.95	1.00
SM.a.C43.2	0.56	1.00
SM.a.C44.6	0.52	1.00
SM.e.C36.2	0.60	1.00
SM.e.C38.3	0.33	1.00
SM.e.C40.5	0.41	1.00

Carn.a.C2.0	0.18	1.00
Carn.a.C3.0	0.92	1.00
Carn.a.C3.0.DC	0.70	1.00
Carn.a.C4.0	0.54	1.00
Carn.a.C5.0	0.14	1.00
Carn.a.C6.0	0.26	1.00
Carn.a.C6.0.OH	0.89	1.00
Carn.a.C8.0	0.23	1.00
Carn.a.C8.1	0.51	1.00
Carn.a.C9.0	0.82	1.00
Carn.a.C10.0	0.19	1.00
Carn.a.C10.1	0.84	1.00
Carn.a.C12.0	0.91	1.00
Carn.a.C14.1	0.89	1.00
Carn.a.C14.2	0.94	1.00
Carn.a.C15.0	0.73	1.00
Carn.a.C16.0	0.34	1.00
Carn.a.C16.0.Oxo	0.51	1.00
Carn.a.C16.1	0.82	1.00
Carn.a.C16.2	0.38	1.00
Carn.a.C18.0	0.78	1.00
Carn.a.C18.1	0.48	1.00
Carn.a.C18.2	0.26	1.00
Carn.a.C18.2.OH	0.44	1.00
Carn.a.C20.0	0.91	1.00
Carn.a.C20.1	0.98	1.00
Carn.a.C20.3	0.36	1.00
Carn.a.C20.4	0.62	1.00
BCAA	0.30	1.00
AAA	0.32	1.00
EAA	0.31	1.00
NEAA	0.21	1.00
AA	0.22	1.00
SATURATEDNEFA	0.03	0.69
MONOUNSATNEFA	0.04	0.69
POLYUNSATNEFA	0.06	0.69
NEFA	0.03	0.69
SATURATEDPCaa	0.98	1.00
MONOUNSATPCaa	0.94	1.00
POLYUNSATPCaa	0.84	1.00
PCaa	0.86	1.00
SATURATEDPCae	0.60	1.00

MONOUNSATPCae	0.58	1.00
POLYUNSATPCae	0.99	1.00
PCae	0.98	1.00
SATURATEDLYSOPCa	0.32	1.00
MONOUNSATLYSOPCa	0.62	1.00
POLYUNSATLYSOPCa	0.67	1.00
LYSOPCa	0.56	1.00
SATURATEDLYSOPCe	0.96	1.00
lyso.PC.e.C18.1	1.00	1.00
LYSOPCe	0.98	1.00
MONOUNSATSM	0.79	1.00
POLYUNSATSM	0.77	1.00
SM	0.77	1.00
Carn	0.65	1.00
SHORTCHAINCARNa	0.24	1.00
MEDIUMCHAINCARNa	0.55	1.00
LONGCHAINCARNa	0.51	1.00
ACYLCARN	0.32	1.00
Asn_Asp	0.78	1.00
Gln_Glu	0.94	1.00
NEFA_18_1_NEFA_18_0	0.76	1.00
NEFA_16_1_NEFA_16_0	0.87	1.00
PC.aa_PC.ae	0.71	1.00
Lyso.PC.a_Lyso.PC.aa	0.38	1.00
LysoPC.a.C16.0C18.0_PCaa	0.21	1.00
LysoPC.a.C18.1C18.2_PCaa	0.94	1.00
Carn.a.C16.0_freeCarn	0.24	1.00
Carn.a.C2.0_Carn.a.C.16.0	0.03	0.69
Val_PCae.C.32.2	0.28	1.00

Supplementary Figure S1.

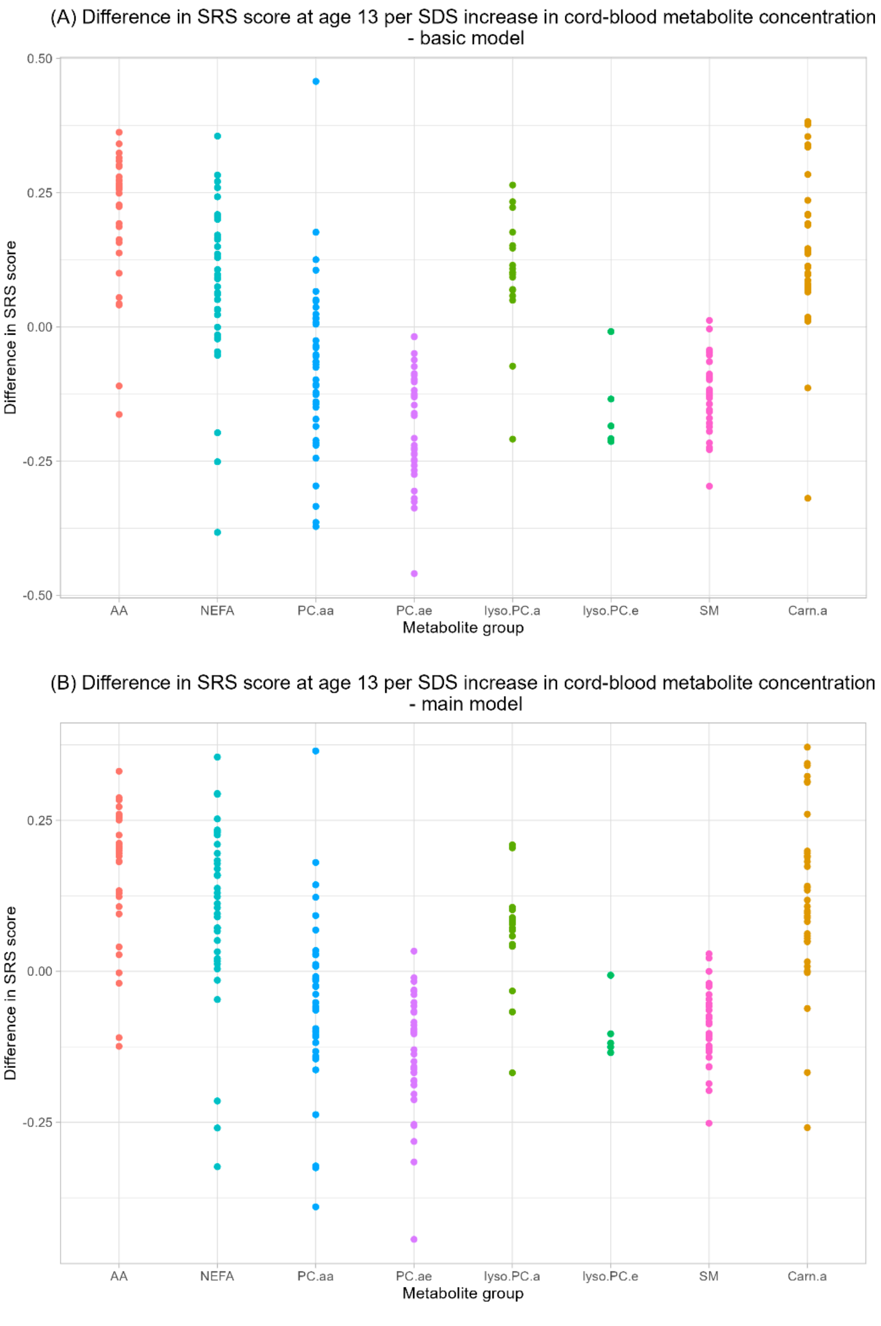

Supplementary Figure S1. Associations of individual cord-blood metabolites with SRS scores at the age of 13 years. (A) basic model, (B) adjusted model. Values represent the estimated change in the (A) is adjusted for sex and age at outcome, (B) adjusted for sex, age at outcome, maternal BMI, maternal psychopathologies, education level, smoking during pregnancy, alcohol intake during pregnancy, gestational age at birth, birthweight. Labeled values represent significant associations (FDR-adjusted p-values < 0.05). Corresponding numerical values are shown in Table S4. AA amino acids, NEFA non-esterified fatty acids, PC.aa diacyl-phosphatidylcholines, PC.ae acyl-alkyl-phosphatidylcholines, lyso.PC.a. acyl-lysophosphatidylcholines, lyso.PC.e alkyl-lysophosphatidylcholines, Carn.a acylcarnitines, SM sphingomyelines.

Supplemental Text S1. Methods for cord blood metabolite measurements

A targeted metabolomics approach was adopted to determine serum concentrations (µmol/L) of AA, NEFA, PL and Carn, as described previously (1). Proteins of 50 µL serum were precipitated by adding 450 µL methanol including internal standards: labeled amino acid standards set A (NSK-A-1, Cambridge Isotope Laboratories (CIL), USA), 15N2-L-asparagine (NLM-3286-0.25, CIL, USA), indole-D5-L-tryptophan (DLM-1092-0.5, CIL, USA), U-13C16-palmitic acid (CLM-409-MPT-PK, CIL, USA), D3-acetyl-carnitine (DLM-754-PK, CIL, USA), D3-octanoyl-carnitine (DLM-755-0.01, CIL, USA) and D3-palmitoyl-carnitine (DLM-1263-0.01, CIL, USA), tridecanoyl-2-hydroxy-sn-glycero-3-phosphocholine (855476, Avanti Polar Lipids, USA) and 1,2-dimyristoyl-sn-glycero-3-phosphocholine (850345, Avanti Polar Lipids, USA). If sample volume was less than optimal, the concentrations were corrected by the respective factor. Sample volumes less than 25 µL were not used and considered missing. After centrifugation the supernatant was split into aliquots. AA were analyzed by liquid chromatography tandem mass spectrometry (LC-MS/MS) as described previously (2). An aliquot of the supernatant was used for the derivatization to AA butylester with hydrochloric acid in 1-butanol. After evaporation, the residues were dissolved in water/methanol (80:20; (v/v)) with 0.1% formic acid. The samples were analyzed with 1100 high-performance liquid chromatography (HPLC) system (Agilent, Waldbronn, Germany) equipped with 150 x 2.1 mm, 3.5 µm particle size C18 HPLC column (X-Bridge, Waters, Milford, USA) and 0.1% heptafluorobutyric acid as and ion pair reagent in the mobile phases A and B (A: water, B: methanol). Mass spectrometric (MS) detection was performed with an API2000 tandem mass spectrometer (AB Sciex, Darmstadt, Germany) equipped with an atmospheric pressure chemical ionization (APCI) source operating in positive ion ionization mode. IUPAC-IUB Nomenclature was used for notation of the AA (1984).

NEFA, PL and Carn were measured with a 1200 SL HPLC system (Agilent, Waldbronn, Germany) coupled to a 4000QTRAP tandem mass spectrometer from AB Sciex (Darmstadt, Germany) (Hellmuth et al. 2012; Uhl et al. 2016)(3, 4). NEFA were analyzed by injection of the supernatant to a LC-MS/MS operating in negative electrospray ionization (ESI) mode where they were separated by gradient elution on a 100 x 3.0 mm, 1.9 µm particle size Purusuit UPS Diphenyl column from Varian (Darmstadt, Germany) using 5 mM ammonium acetate in water as mobile phase A and acetonitrile/ isopropanol (80:20; (v/v)) as mobile phase B. NEFA species were quantified using GLC-85 reference standard mixture (Nu-Chek Prep, USA). PL were analyzed by flow-injection analysis (FIA) with LC-MS/MS coupled with ESI (Rauschert et al. 2016)(5). The system was run in positive ionization mode with 5% water in isopropanol as mobile phase A and 5% water in methanol as mobile phase B. The

analysis was performed for diacyl-phosphatidylcholines (PC.aa), acyl-alkyl-phosphatidylcholines (PC.ae) , acyl-lysophosphatidylcholines (Lyso.PC.a), alkyl-lysophosphatidylcholines (Lyso.PC.e) and sphingomyelines (SM)). Carn (Free carnitine (Free Carn) and acyl-carnitines (Carn.a)) were analyzed by flow-injection analysis of the supernatant into a LC-MS/MS system using an isocratic elution with 76% isopropanol, 19% methanol and 5% water. The mass spectrometer was equipped with electrospray ionization and operated in positive ionization mode. PL and acyl-carn were quantified using aliquots of a commercial available lyophilized control plasma (ClinChek®, Recipe, Germany), where the concentrations have been determined by AbsoluteIDQ p150 Kit from Biocrates®, a previous published LC-MS/MS method (Uhl et al. 2011)(6) and by in-house quantification with various standards. The analytical technique used is capable of determining the total number of total bonds, but not the position of the double bonds and the distribution of the carbon atoms between fatty acid side chains. We used the following notation for NEFA, PL and Carn.a: X:Y, where X denotes the length of the carbon chain, and Y the number of double bonds. The 'a' denotes an acyl chain bound to the backbone via an ester bond ('acyl-') and the 'e' represents an ether bond ('alkyl-').

Supplemental Table S10. Parameters for mass-spectrometry detection and identifications

Parameters for mass-spectrometry detection and identification for amino-acids and non-esterified fatty acids, including the labelled internal standards .

ID*	Rt minutes	Q1	Q3	DP	CE	CXP	Adduct	MSI ID Level
**Amino acids**								
Ala1	7,6	146,182	44	11	25	4	(M+H)+	1
Ala2	7,6	146,182	90	11	13	12	(M+H)+	1
AlaIS	7,6	150,168	48,1	31	25	6	(M+H)+	1
Arg1	7,3	231,201	70,1	21	39	8	(M+H)+	1
Arg2	7,3	231,276	60	21	33	8	(M+H)+	1
Arg3	7,3	231,276	172,2	21	21	8	(M+H)+	1
ArgIS	7,3	236,201	75,1	21	39	8	(M+H)+	1
Asn1	5,4	189,303	144,1	21	17	6	(M+H)+	1
Asn2	5,4	189,303	74	21	27	8	(M+H)+	1
Asn3	5,4	189,303	130,3	21	19	4	(M+H)+	1
AsnIS	5,4	191,116	145,2	21	19	6	(M+H)+	1
Asp1	13,8	246,262	144,3	21	19	6	(M+H)+	1
Asp2	13,8	246,262	88,1	21	27	2	(M+H)+	1
Asp3	13,8	246,262	74,2	21	35	8	(M+H)+	1
AspIS	13,8	249,278	147,3	16	19	6	(M+H)+	1
Cit1	6,3	232,249	70,1	16	43	8	(M+H)+	1
Cit2	6,3	232,249	215,3	16	17	10	(M+H)+	1
Cit3	6,3	232,249	113,2	16	27	4	(M+H)+	1
CitIS	6,3	234,237	115,2	16	27	4	(M+H)+	1

Cys1	11,8	353,133	129,9	21	29	14	(M+H)+	1
Cys2	11,8	353,078	73,9	21	47	8	(M+H)+	1
Cys3	11,8	353,078	208,1	21	21	8	(M+H)+	1
CysIS	11,8	357,133	129,9	21	29	14	(M+H)+	1
Gln1	5,6	203,1	84,1	11	30	11	(M+H)+	1
Gln2	5,6	203,1	186,1	11	16	8	(M+H)+	1
Gln3	5,6	203,1	130,1	11	21	15	(M+H)+	1
GlnIS	5,6	208,1	89,1	11	30	11	(M+H)+	1
Glu1	14,3	260,312	84	16	37	10	(M+H)+	1
Glu2	14,3	260,312	186,2	16	19	8	(M+H)+	1
Glu3	14,3	260,312	130,1	16	25	4	(M+H)+	1
GluIS	14,3	263,297	87,1	16	35	10	(M+H)+	1
Gly1	6,2	132,19	76	16	13	8	(M+H)+	1
Gly2	6,2	132,19	57	16	19	6	(M+H)+	1
GlyIS	6,2	134,17	77,9	16	13	10	(M+H)+	1
His1	6,8	212,18	109,9	16	27	14	(M+H)+	1
His2	6,8	212,271	83,1	21	41	10	(M+H)+	1
His3	6,8	212,271	93	21	41	8	(M+H)+	1
HisIS	6,8	215,184	112,9	16	27	14	(M+H)+	1
Ile1	12,4	188,327	86	21	21	10	(M+H)+	1
Ile2	12,4	188,327	69	21	33	8	(M+H)+	1
Ile3	12,4	188,327	44,1	21	43	4	(M+H)+	1
Leu1	12,6	188,2	86	21	21	10	(M+H)+	1
Leu2	12,6	188,2	69	21	33	8	(M+H)+	1
Leu3	12,6	188,2	44,1	21	43	4	(M+H)+	1
LeuIS	12,6	191,338	89,2	11	19	2	(M+H)+	1
Lys1	7,2	203,2	84,1	21	33	2	(M+H)+	1
Lys2	7,2	203,2	186,2	21	17	8	(M+H)+	1
Lys3	7,2	203,2	56	21	61	6	(M+H)+	1
LysIS	7,2	207,2	88,1	21	33	2	(M+H)+	1
Met1	10,9	206,245	104,1	31	19	4	(M+H)+	1
Met2	10,9	206,245	61,1	31	41	6	(M+H)+	1
Met3	10,9	206,245	56	31	31	6	(M+H)+	1
MetIS	10,9	209,2	107,1	11	30	5	(M+H)+	1
Orn1	6,7	189,304	70,1	16	29	8	(M+H)+	1
Orn2	6,7	189,304	172,2	16	15	8	(M+H)+	1
Orn3	6,7	189,304	116,1	16	21	6	(M+H)+	1
OrnIS	6,7	191,338	174,1	11	15	8	(M+H)+	1
Phe1	12,8	222,248	120,3	21	23	4	(M+H)+	1
Phe2	12,8	222,248	103,1	21	49	10	(M+H)+	1
Phe3	12,8	222,248	77	21	69	8	(M+H)+	1
PheIS	12,8	228,284	126,2	16	21	6	(M+H)+	1
Pro1	7,8	172,291	70,1	26	25	8	(M+H)+	1

Pro2	7,8	172,291	116,2	26	19	4	(M+H)+	1
Pro3	7,8	172,291	57,1	26	27	6	(M+H)+	1
ProIS	7,8	175,18	73	16	27	8	(M+H)+	1
Pro2IS	7,8	175,18	118,9	16	21	16	(M+H)+	1
Ser1	6	162,255	60	16	23	6	(M+H)+	1
Ser2	6	162,255	106,2	16	15	4	(M+H)+	1
Ser3	6	162,255	88,3	16	19	2	(M+H)+	1
SerIS	6	165,255	63	16	23	6	(M+H)+	1
Thr1	7,2	176,24	73,9	16	23	10	(M+H)+	1
Thr2	7,2	176,24	55,9	16	31	6	(M+H)+	1
Thr3	7,2	176,24	102,1	16	19	4	(M+H)+	1
ThrIS	7,2	180,24	75,9	16	23	10	(M+H)+	1
Trp1	13,3	261,284	244,2	21	17	10	(M+H)+	1
Trp2	13,3	261,284	159,3	21	25	6	(M+H)+	1
Trp3	13,3	261,284	132,4	21	41	6	(M+H)+	1
TrpIS	13,3	266,284	249,2	21	17	10	(M+H)+	1
Tyr1	10,24	238,241	136,1	21	23	6	(M+H)+	1
Tyr2	10,24	238,241	91,2	21	47	4	(M+H)+	1
Tyr3	10,24	238,241	119,2	21	37	4	(M+H)+	1
TyrIS	10,24	244,266	142,2	16	23	8	(M+H)+	1
Val1	10,8	174,213	72	16	19	8	(M+H)+	1
Val2	10,8	174,213	55	16	41	6	(M+H)+	1
Val3	10,8	174,213	118,2	16	15	6	(M+H)+	1
ValIS	10,8	182,275	80,2	16	21	10	(M+H)+	1
**Non-esterified fatty acids**								
4_0	0,7	87	87	-45	-8	-7	(M-H)-	1
5_0	0,8	101	101	-45	-8	-7	(M-H)-	1
6_0	1,0	115,1	115,1	-50	-8	-7	(M-H)-	1
7_0	1,2	129,1	129,1	-50	-8	-7	(M-H)-	1
8_0	1,6	143,1	143,1	-55	-8	-7	(M-H)-	1
9_0	2,0	157,1	157,1	-55	-8	-7	(M-H)-	1
10_0	2,4	171,146	171,146	-60	-8	-13	(M-H)-	1
11_0	2,7	185,162	185,162	-65	-8	-11	(M-H)-	1
12_0	3,1	199,178	199,178	-68	-8	-11	(M-H)-	1
12_1	2,6	197,162	197,162	-72	-8	-7	(M-H)-	1
13_0	3,5	213,193	213,193	-70	-8	-17	(M-H)-	1
13_1	2,9	211,178	211,178	-74	-8	-7	(M-H)-	1
14_0	3,9	227,209	227,209	-120	-25	-13	(M-H)-	1
14_1	3,3	225,193	225,193	-75	-8	-13	(M-H)-	1
14_2	2,7	223,178	223,178	-78	-8	-7	(M-H)-	1
15_0	4,2	241,225	241,225	-75	-8	-7	(M-H)-	1
15_1	3,7	239,209	239,209	-75	-10	-7	(M-H)-	1
16_0	4,6	255,24	255,24	-150	-35	-13	(M-H)-	1

16_1	4,0	253,225	253,225	-78	-30	-13	(M-H)-	1
16_2	3,5	251,209	251,209	-79	-8	-7	(M-H)-	1
16_3	2,9	249,193	249,193	-78	-8	-7	(M-H)-	1
16_4	2,4	247,178	247,178	-78	-8	-7	(M-H)-	1
17_0	5,0	269,256	269,256	-85	-12	-7	(M-H)-	1
17_1	4,4	267,24	267,24	-75	-10	-7	(M-H)-	1
17_2	3,9	265,225	265,225	-79	-8	-7	(M-H)-	1
18_0	5,4	283,272	283,272	-150	-35	-7	(M-H)-	1
18_1	4,8	281,256	281,256	-150	-37	-7	(M-H)-	1
18_2	4,2	279,24	279,24	-130	-32	-7	(M-H)-	1
18_3	3,7	277,225	277,225	-120	-10	-7	(M-H)-	1
18_4	3,1	275,209	275,209	-72	-8	-7	(M-H)-	1
19_0	5,7	297,287	297,287	-90	-8	-7	(M-H)-	1
19_1	5,2	295,272	295,272	-85	-8	-7	(M-H)-	1
19_2	4,6	293,256	293,256	-80	-8	-7	(M-H)-	1
20_0	6,1	311,303	311,303	-95	-8	-13	(M-H)-	1
20_1	5,5	309,287	309,287	-90	-8	-9	(M-H)-	1
20_2	5,0	307,272	307,272	-85	-8	-9	(M-H)-	1
20_3	4,4	305,256	305,256	-80	-8	-9	(M-H)-	1
20_4	3,9	303,24	303,24	-150	-10	-9	(M-H)-	1
20_5	3,3	301,225	301,225	-61	-8	-7	(M-H)-	1
22_0	6,8	339,334	339,334	-100	-14	-11	(M-H)-	1
22_1	6,3	337,318	337,318	-80	-8	-9	(M-H)-	1
22_2	5,7	335,303	335,303	-80	-8	-11	(M-H)-	1
22_3	5,2	333,287	333,287	-71	-8	-7	(M-H)-	1
22_4	4,6	331,272	331,272	-62	-8	-7	(M-H)-	1
22_5	4,0	329,256	329,256	-53	-8	-7	(M-H)-	1
22_6	3,5	327,24	327,24	-150	-6	-13	(M-H)-	1
24_0	7,6	367,365	367,365	-106	-8	-7	(M-H)-	1
24_1	7,0	365,35	365,35	-97	-8	-11	(M-H)-	1
24_2	6,5	363,334	363,334	-81	-8	-7	(M-H)-	1
24_3	5,9	361,318	361,318	-69	-8	-7	(M-H)-	1
24_4	5,4	359,303	359,303	-57	-8	-7	(M-H)-	1
24_5	4,8	357,287	357,287	-44	-8	-7	(M-H)-	1
24_6	4,2	355,272	355,272	-32	-8	-7	(M-H)-	1
26_0	8,3	395,397	395,397	-113	-8	-7	(M-H)-	1
26_1	7,8	393,381	393,381	-97	-8	-7	(M-H)-	1
26_2	7,2	391,365	391,365	-82	-8	-7	(M-H)-	1
26_3	6,7	389,35	389,35	-67	-8	-7	(M-H)-	1
26_4	6,1	387,334	387,334	-52	-8	-7	(M-H)-	1
26_5	5,5	385,318	385,318	-36	-8	-7	(M-H)-	1
26_6	5,0	383,303	383,303	-21	-8	-7	(M-H)-	1
16_0-IS	4,1	271	271	-80	-10	-7	(M-H)-	1

20_4-IS	3,7	311,24	311,24	-70	-10	-9	(M-H)-	1
22_6-IS	3,6	332,24	332,24	-40	-6	-13	(M-H)-	1
22_0-IS	5,9	342,33	342,33	-100	-14	-11	(M-H)-	1
10_0-IS	2,4	190,15	190,15	-60	-8	-13	(M-H)-	1
6_0-IS	0,9	126,1	126,1	-50	-8	-7	(M-H)-	1

ID metabolite identity, Rt retention time in minutes, Q1/Q3 quadrupole 1 and 3, IS internal standard, DP declustering potential, CE collison energy, CXP collision cell exit potential, MSI ID Metabolomics Standards Initiative identification.
*The numbers next to the ID refer to the different transitions used.

Parameters for mass-spectrometry detection and identifications for phospholipids and acyl-carnitines.

**ID**	**Sofia.ID**	**Q1**	**Q3**	**CP1**	**CP2**	**Adduct**	**MSI ID Level**
Carn.C0	Carn	162,1	85,1	29,25	29,85	(M+H)+	1
Carn.C10	Carn.C10	316,2	85,1	0,1465	0,1395	(M+H)+	1
Carn.C10.1	Carn.C10.1	314,2	85,1	0,13	0,122	(M+H)+	1
Carn.C10.2	Carn.C10.2	312,2	85,1	0,0275	0,0275	(M+H)+	1
Carn.C12	Carn.C12	344,3	85,1	0,06975	0,0705	(M+H)+	1
Carn.C12.1	Carn.C12.1	342,3	85,1	0,13875	0,1245	(M+H)+	1
Carn.C12.DC	Carn.C12.DC	374,3	85,1	0,0475	0,0495	(M+H)+	1
Carn.C14	Carn.C14	372,3	85,1	0,03875	0,037	(M+H)+	1
Carn.C14.1	Carn.C14.1	370,3	85,1	0,07725	0,078	(M+H)+	1
Carn.C14.1.OH	Carn.C14.1.OH	386,3	85,1	0,0095	0,0095	(M+H)+	1
Carn.C14.2	Carn.C14.2	368,3	85,1	0,014	0,0135	(M+H)+	1
Carn.C14.2.OH	Carn.C14.2.OH	384,3	85,1	0,0075	0,007	(M+H)+	1
Carn.C16	Carn.C16	400,3	85,1	0,075	0,0775	(M+H)+	1
Carn.C16.1	Carn.C16.1	398,3	85,1	0,02625	0,027	(M+H)+	1
Carn.C16.1.OH	Carn.C16.1.OH	414,3	85,1	0,01	0,0095	(M+H)+	1
Carn.C16.2	Carn.C16.2	396,3	85,1	0,0045	0,005	(M+H)+	1
Carn.C16.2.OH	Carn.C16.2.OH	412,3	85,1	0,01	0,0095	(M+H)+	1
Carn.C16.OH	Carn.C16.OH	416,3	85,1	0,0055	0,0055	(M+H)+	1
Carn.C18	Carn.C18	428,4	85,1	0,03425	0,0355	(M+H)+	1
Carn.C18.1	Carn.C18.1	426,4	85,1	0,0915	0,0915	(M+H)+	1
Carn.C18.1.OH	Carn.C18.1.OH	442,4	85,1	0,0075	0,0075	(M+H)+	1
Carn.C18.2	Carn.C18.2	424,3	85,1	0,043	0,043	(M+H)+	1
Carn.C2	Carn.C2	204,1	85,1	4,48	4,52	(M+H)+	1
Carn.C3	Carn.C3	218,1	85,1	0,345	0,355	(M+H)+	1
Carn.C3.1	Carn.C3.1	216,1	85,1	0,0055	0,005	(M+H)+	1
Carn.C3.DC.C4.OH.	Carn.C3.DC	248,1	85,1	0,03875	0,0495	(M+H)+	1
Carn.C3.OH	Carn.C3.OH	234,1	85,1	0,02125	0,022	(M+H)+	1
Carn.C4	Carn.C4	232,2	85,1	0,179	0,1875	(M+H)+	1
Carn.C4.1	Carn.C4.1	230,1	85,1	0,019	0,019	(M+H)+	1
Carn.C5	Carn.C5	246,2	85,1	0,10375	0,103	(M+H)+	1
Carn.C5.1	Carn.C5.1	244,2	85,1	0,0195	0,02	(M+H)+	1
Carn.C5.1.DC	Carn.C5.1.DC	274,1	85,1	0,0175	0,015	(M+H)+	1

Carn.C5.DC.C6.OH.	Carn.C5.DC	276,1	85,1	0,01925	0,0205	(M+H)+	1
Carn.C5.M.DC	Carn.C5.M.DC	290,2	85,1	0,03225	0,031	(M+H)+	1
Carn.C5.OH.C3.DC.M.	Carn.C5.OH	262,2	85,1	0,0575	0,0615	(M+H)+	1
Carn.C6.1	Carn.C6.1	258,2	85,1	0,0125	0,0125	(M+H)+	1
Carn.C6.C4.1.DC.	Carn.C6	260,2	85,1	0,04475	0,046	(M+H)+	1
Carn.C7.DC	Carn.C7.DC	304,2	85,1	0,02575	0,027	(M+H)+	1
Carn.C8	Carn.C8	288,2	85,1	0,1105	0,1195	(M+H)+	1
Carn.C8.1	Carn.C8.1	286,2	85,1	0,07525	0,0745	(M+H)+	1
Carn.C9	Carn.C9	302,2	85,1	0,03425	0,034	(M+H)+	1
lysoPCaC14.0	lyso.PC.a.C14.0	468,3	184	2,575	2,615	(M+H)+	1
lysoPCaC16.0	lyso.PC.a.C16.0	496,3	184	98,775	95,6	(M+H)+	1
lysoPCaC16.1	lyso.PC.a.C16.1	494,3	184	3	2,96	(M+H)+	1
lysoPCaC17.0	lyso.PC.a.C17.0	510,4	184	1,7325	1,66	(M+H)+	1
lysoPCaC18.0	lyso.PC.a.C18.0	524,4	184	26,675	26,2	(M+H)+	1
lysoPCaC18.1	lyso.PC.a.C18.1	522,4	184	18,275	18,05	(M+H)+	1
lysoPCaC18.2	lyso.PC.a.C18.2	520,3	184	32,6	32,15	(M+H)+	1
lysoPCaC20.3	lyso.PC.a.C20.3	546,4	184	1,96	2,03	(M+H)+	1
lysoPCaC20.4	lyso.PC.a.C20.4	544,3	184	6,5375	6,48	(M+H)+	1
lysoPCaC24.0	lyso.PC.a.C24.0	608,5	184	0,76625	0,8805	(M+H)+	1
lysoPCaC26.0	lyso.PC.a.C26.0	636,5	184	1,815	2,22	(M+H)+	1
lysoPCaC26.1	lyso.PC.a.C26.1	634,5	184	3,4275	3,71	(M+H)+	1
lysoPCaC28.0	lyso.PC.a.C28.0	664,5	184	1,3125	1,535	(M+H)+	1
lysoPCaC28.1	lyso.PC.a.C28.1	662,5	184	1,52	1,785	(M+H)+	1
lysoPCaC6.0	lyso.PC.a.C6.0	356,2	184	0,05025	0,0455	(M+H)+	1
PCaaC24.0	PC.aa.C24.0	622,4	184	0,4935	0,596	(M+H)+	1
PCaaC26.0	PC.aa.C26.0	650,5	184	2,735	3,33	(M+H)+	1
PCaaC28.1	PC.aa.C28.1	676,5	184	2,6525	2,775	(M+H)+	1
PCaaC30.0	PC.aa.C30.0	706,5	184	3,49	3,535	(M+H)+	1
PCaaC30.2	PC.aa.C30.2	702,5	184	0,51625	0,548	(M+H)+	1
PCaaC32.0	PC.aa.C32.0	734,6	184	11	11,05	(M+H)+	1
PCaaC32.1	PC.aa.C32.1	732,6	184	13,3	13,75	(M+H)+	1
PCaaC32.2	PC.aa.C32.2	730,5	184	2,8125	2,87	(M+H)+	1
PCaaC32.3	PC.aa.C32.3	728,5	184	0,5415	0,525	(M+H)+	1
PCaaC34.1	PC.aa.C34.1	760,6	184	167,5	167,5	(M+H)+	1
PCaaC34.2	PC.aa.C34.2	758,6	184	278,75	282	(M+H)+	1
PCaaC34.3	PC.aa.C34.3	756,6	184	14,65	13,6	(M+H)+	1
PCaaC34.4	PC.aa.C34.4	754,5	184	1,675	1,65	(M+H)+	1
PCaaC36.0	PC.aa.C36.0	790,6	184	2,305	2,095	(M+H)+	1
PCaaC36.1	PC.aa.C36.1	788,6	184	35,4	34,15	(M+H)+	1
PCaaC36.2	PC.aa.C36.2	786,6	184	170,5	173	(M+H)+	1
PCaaC36.3	PC.aa.C36.3	784,6	184	97,8	98,9	(M+H)+	1
PCaaC36.4	PC.aa.C36.4	782,6	184	125	127	(M+H)+	1
PCaaC36.5	PC.aa.C36.5	780,6	184	15,975	15,75	(M+H)+	1

PCaaC36.6	PC.aa.C36.6	778,5	184	0,9525	0,848	(M+H)+	1
PCaaC38.0	PC.aa.C38.0	818,7	184	1,8325	1,81	(M+H)+	1
PCaaC38.1	PC.aa.C38.1	816,6	184	1,17	0,9875	(M+H)+	1
PCaaC38.3	PC.aa.C38.3	812,6	184	29,275	28,55	(M+H)+	1
PCaaC38.4	PC.aa.C38.4	810,6	184	69,225	72,3	(M+H)+	1
PCaaC38.5	PC.aa.C38.5	808,6	184	34,775	35,8	(M+H)+	1
PCaaC38.6	PC.aa.C38.6	806,6	184	47,4	49,6	(M+H)+	1
PCaaC40.1	PC.aa.C40.1	844,7	184	0,457	0,425	(M+H)+	1
PCaaC40.2	PC.aa.C40.2	842,7	184	0,51675	0,436	(M+H)+	1
PCaaC40.3	PC.aa.C40.3	840,6	184	0,72825	0,646	(M+H)+	1
PCaaC40.4	PC.aa.C40.4	838,6	184	2,4325	2,51	(M+H)+	1
PCaaC40.5	PC.aa.C40.5	836,6	184	6,835	7,19	(M+H)+	1
PCaaC40.6	PC.aa.C40.6	834,6	184	15,6	16,6	(M+H)+	1
PCaaC42.0	PC.aa.C42.0	874,7	184	0,4535	0,4445	(M+H)+	1
PCaaC42.1	PC.aa.C42.1	872,7	184	0,2585	0,245	(M+H)+	1
PCaaC42.2	PC.aa.C42.2	870,7	184	0,2725	0,233	(M+H)+	1
PCaaC42.4	PC.aa.C42.4	866,7	184	0,248	0,222	(M+H)+	1
PCaaC42.5	PC.aa.C42.5	864,6	184	0,3165	0,3175	(M+H)+	1
PCaaC42.6	PC.aa.C42.6	862,6	184	0,55475	0,58	(M+H)+	1
PCaeC30.0	PC.ae.C30.0	692,6	184	0,432	0,4415	(M+H)+	1
PCaeC30.1	PC.ae.C30.1	690,5	184	0,691	0,882	(M+H)+	1
PCaeC30.2	PC.ae.C30.2	688,5	184	0,249	0,2505	(M+H)+	1
PCaeC32.1	PC.ae.C32.1	718,6	184	2,2625	2,28	(M+H)+	1
PCaeC32.2	PC.ae.C32.2	716,6	184	0,798	0,8295	(M+H)+	1
PCaeC34.0	PC.ae.C34.0	748,6	184	1,3025	1,275	(M+H)+	1
PCaeC34.1	PC.ae.C34.1	746,6	184	7,3125	7,285	(M+H)+	1
PCaeC34.2	PC.ae.C34.2	744,6	184	8,33	8,28	(M+H)+	1
PCaeC34.3	PC.ae.C34.3	742,6	184	5,845	6,035	(M+H)+	1
PCaeC36.0	PC.ae.C36.0	776,7	184	0,765	0,7355	(M+H)+	1
PCaeC36.1	PC.ae.C36.1	774,6	184	7,6675	6,57	(M+H)+	1
PCaeC36.2	PC.ae.C36.2	772,6	184	11,525	10,75	(M+H)+	1
PCaeC36.3	PC.ae.C36.3	770,6	184	5,5775	5,515	(M+H)+	1
PCaeC36.4	PC.ae.C36.4	768,6	184	11,05	11,15	(M+H)+	1
PCaeC36.5	PC.ae.C36.5	766,6	184	7,2275	7,405	(M+H)+	1
PCaeC38.0	PC.ae.C38.0	804,7	184	2,195	2,045	(M+H)+	1
PCaeC38.1	PC.ae.C38.1	802,7	184	1,8875	1,41	(M+H)+	1
PCaeC38.2	PC.ae.C38.2	800,7	184	3,125	2,535	(M+H)+	1
PCaeC38.3	PC.ae.C38.3	798,6	184	5,1625	4,68	(M+H)+	1
PCaeC38.4	PC.ae.C38.4	796,6	184	9,3975	9,28	(M+H)+	1
PCaeC38.5	PC.ae.C38.5	794,6	184	10,525	10,55	(M+H)+	1
PCaeC38.6	PC.ae.C38.6	792,6	184	4,71	4,805	(M+H)+	1
PCaeC40.0	PC.ae.C40.0	832,7	184	7,225	7,365	(M+H)+	1
PCaeC40.1	PC.ae.C40.1	830,7	184	1,555	1,55	(M+H)+	1

PCaeC40.2	PC.ae.C40.2	828,7	184	1,7025	1,6	(M+H)+	1
PCaeC40.3	PC.ae.C40.3	826,7	184	1,65	1,45	(M+H)+	1
PCaeC40.4	PC.ae.C40.4	824,7	184	2,0575	1,955	(M+H)+	1
PCaeC40.5	PC.ae.C40.5	822,6	184	3,285	3,24	(M+H)+	1
PCaeC40.6	PC.ae.C40.6	820,6	184	2,885	2,93	(M+H)+	1
PCaeC42.0	PC.ae.C42.0	860,7	184	0,52825	0,533	(M+H)+	1
PCaeC42.1	PC.ae.C42.1	858,7	184	0,53275	0,5395	(M+H)+	1
PCaeC42.2	PC.ae.C42.2	856,7	184	0,54675	0,5235	(M+H)+	1
PCaeC42.3	PC.ae.C42.3	854,7	184	1,065	0,9655	(M+H)+	1
PCaeC42.4	PC.ae.C42.4	852,7	184	0,83025	0,7745	(M+H)+	1
PCaeC42.5	PC.ae.C42.5	850,7	184	1,765	1,76	(M+H)+	1
PCaeC44.3	PC.ae.C44.3	882,7	184	0,28225	0,219	(M+H)+	1
PCaeC44.4	PC.ae.C44.4	880,7	184	0,4375	0,412	(M+H)+	1
PCaeC44.5	PC.ae.C44.5	878,7	184	1,4725	1,51	(M+H)+	1
PCaeC44.6	PC.ae.C44.6	876,7	184	0,85425	0,8785	(M+H)+	1
SM.OH.C14.1	SM.C18.1.OH.C14.1	689,5	184	4,4825	4,26	(M+H)+	1
SM.OH.C16.1	SM.C18.1.OH.C16.1	717,6	184	2,065	2,075	(M+H)+	1
SM.OH.C22.1	SM.C18.1.OH.C22.1	801,6	184	8,58	8,365	(M+H)+	1
SM.OH.C22.2	SM.C18.1.OH.C22.2	799,6	184	6,26	6,14	(M+H)+	1
SM.OH.C24.1	SM.C18.1.OH.C24.1	829,7	184	0,9405	0,9585	(M+H)+	1
SMC16.0	SM.C18.1.C16.0	703,6	184	76,4	75	(M+H)+	1
SMC16.1	SM.C18.1.C16.1	701,6	184	10,525	10,55	(M+H)+	1
SMC18.0	SM.C18.1.C18.0	731,6	184	14,575	14,35	(M+H)+	1
SMC18.1	SM.C18.1.C18.1	729,6	184	7,1225	7,025	(M+H)+	1
SMC20.2	SM.C18.1.C20.2	755,6	184	0,322	0,282	(M+H)+	1
SMC22.3	SM.C18.1.C22.3	781,6	184	1,7625	1,755	(M+H)+	1
SMC24.0	SM.C18.1.C24.0	815,7	184	14,8	14,3	(M+H)+	1
SMC24.1	SM.C18.1.C24.1	813,7	184	33	32,15	(M+H)+	1
SMC26.0	SM.C18.1.C26.0	843,7	184	0,07225	0,0815	(M+H)+	1
SMC26.1	SM.C18.1.C26.1	841,7	184	0,28175	0,2855	(M+H)+	1
Sum of Hexoses	Sum of Hexoses	179	89	22237,5	22592	(M+H)+	1

ID metabolite identity metabolomics laboratory. Sofia ID metabolite identity Generation R Study group. Q1/Q3 Quadrupole 1 and 3, CP1/CP2 Calibrators 1 and 2, MSI ID Metabolomics Standards Initiative identification.
This table is adapted from: Voerman, E., Jaddoe, V. W. V., Uhl, O., & Shokry, E. (2020). A population based resource for intergenerational metabolomics analysis in pregnant women and their children : the Generation R Study. *Metabolomics*, 1 – 26. Voerman et al. 2020 describes the acquisition, processing and structure of the metabolomics data in the Generation R study cohort. (7)

REFERENCES

1. Hellmuth C, Uhl O, Standl M, Demmelmair H, Heinrich J, Koletzko B, Thiering E. Cord Blood Metabolome Is Highly Associated with Birth Weight, but Less Predictive for Later Weight Development. Obes Facts. 2017;10(2):85-100.
2. Harder U, Koletzko B, Peissner W. Quantification of 22 plasma amino acids combining derivatization and ion-pair LC-MS/MS. J Chromatogr B Analyt Technol Biomed Life Sci. 2011;879(7-8):495-504.
3. Hellmuth C, Weber M, Koletzko B, Peissner W. Nonesterified fatty acid determination for functional lipidomics: comprehensive ultrahigh performance liquid chromatography-tandem mass spectrometry quantitation, qualification, and parameter prediction. Anal Chem. 2012;84(3):1483-90.
4. Uhl O, Fleddermann M, Hellmuth C, Demmelmair H, Koletzko B. Phospholipid Species in Newborn and 4 Month Old Infants after Consumption of Different Formulas or Breast Milk. PLoS One. 2016;11(8):e0162040.
5. Rauschert S, Mori TA, Beilin LJ, Jacoby P, Uhl O, Koletzko B, et al. Early Life Factors, Obesity Risk, and the Metabolome of Young Adults. Obesity (Silver Spring). 2017;25(9):1549-55.
6. Uhl O, Glaser C, Demmelmair H, Koletzko B. Reversed phase LC/MS/MS method for targeted quantification of glycerophospholipid molecular species in plasma. J Chromatogr B Analyt Technol Biomed Life Sci. 2011;879(30):3556-64.
7. Voerman E, Jaddoe VWV, Uhl O, Shokry E, Horak J, Felix JF, et al. A population-based resource for intergenerational metabolomics analyses in pregnant women and their children: the Generation R Study. Metabolomics. 2020;16(4):43.
